# Crosstalk between androgen receptor and WNT/β-catenin signaling causes sex-specific adrenocortical hyperplasia in mice

**DOI:** 10.1242/dmm.050053

**Published:** 2023-05-10

**Authors:** Rodanthi Lyraki, Anaëlle Grabek, Amélie Tison, Lahiru Chamara Weerasinghe Arachchige, Mirko Peitzsch, Nicole Bechmann, Sameh A. Youssef, Alain de Bruin, Elvira R. M. Bakker, Frank Claessens, Marie-Christine Chaboissier, Andreas Schedl

**Affiliations:** ^1^ Université Côte d'Azur, Inserm, CNRS, Institut de Biologie Valrose, 06108 Nice, France; ^2^ Institute of Clinical Chemistry and Laboratory Medicine, University Hospital Carl Gustav Carus, Technische Universität Dresden, Fetscherstrasse 74, 01307 Dresden, Germany; ^3^ Department of Medicine III, University Hospital Carl Gustav Carus, Technische Universität Dresden, Fetscherstrasse 74, 01307 Dresden, Germany; ^4^ Dutch Molecular Pathology Center, Department of Biomolecular Health Sciences, Faculty of Veterinary Medicine, Utrecht University, 3584 CL, Utrecht, the Netherlands; ^5^ Janssen Research and Development, 2340 Beerse, Belgium; ^6^ Department of Pediatrics, University of Groningen, University Medical Center Groningen, 9713 AV, Groningen, the Netherlands; ^7^ Department of Pathology, University Medical Center Utrecht, 3508 AB, Utrecht, the Netherlands; ^8^ Molecular Endocrinology Laboratory, Department of Cellular and Molecular Medicine, KU Leuven, 3000 Leuven, Belgium

**Keywords:** R-spondin signaling, Androgen receptor, Adrenocortical hyperplasia, Sexual dimorphism

## Abstract

Female bias is highly prevalent in conditions such as adrenal cortex hyperplasia and neoplasia, but the reasons behind this phenomenon are poorly understood. In this study, we show that overexpression of the secreted WNT agonist R-spondin 1 (RSPO1) leads to ectopic activation of WNT/β-catenin signaling and causes sex-specific adrenocortical hyperplasia in mice. Although female adrenals show ectopic proliferation, male adrenals display excessive immune system activation and cortical thinning. Using a combination of genetic manipulations and hormonal treatment, we show that gonadal androgens suppress ectopic proliferation in the adrenal cortex and determine the selective regulation of the WNT-related genes *Axin2* and *Wnt4*. Notably, genetic removal of androgen receptor (AR) from adrenocortical cells restores the mitogenic effect of WNT/β-catenin signaling. This is the first demonstration that AR activity in the adrenal cortex determines susceptibility to canonical WNT signaling-induced hyperplasia.

## Teaser

**Summary:** Activation of R-spondin 1 signaling in the adrenal cortex leads to a sexually dimorphic phenotype in mice, causing hyperplasia in females and immune cell recruitment in males.

## INTRODUCTION

Sexual dimorphism is prevalent among mammalian phenotypic traits (Karp et al., 2017) and underlies several aspects of mammalian physiology, including malignant transformation (Clocchiatti et al., 2016) and immunity (Klein and Flanagan, 2016). Sex-specific effects often stem from the action of gonadal hormones (Yu et al., 2020) but can also have sex chromosome-related causes, such as the incomplete inactivation of X chromosome genes (Chen et al., 2012). An important open question is whether sex impacts size maintenance and homeostasis of self-renewing adult tissues, such as the adrenal cortex.

Adrenals are endocrine organs consisting of a non-endocrine capsule surrounding the outer cortex, responsible for the synthesis of steroid hormones, and the inner medulla, responsible for the synthesis of catecholamines. The adrenal cortex is characterized by the expression of the transcription factor SF1 (*Nr5a1*) and is further divided into concentric rings that form a characteristic zonation pattern (Pignatti et al., 2017). The outer zona glomerulosa (zG) produces mineralocorticoids, the middle zona fasciculata (zF) produces glucocorticoids, and the inner zona reticularis (zR) produces androgens. The latter is absent in mice; in its place, we find the X-zone, a transient remnant of the fetal adrenal with unknown functions in adulthood (Huang and Kang, 2019).

The adrenal cortex undergoes constant renewal owing to resident populations of stem/progenitor cells that are primarily concentrated in the capsule and the sub-capsular zG (Ching and Vilain, 2009; Finco et al., 2018; King et al., 2009; Lyraki and Schedl, 2021b). A proliferating zone can be distinguished in the outer cortex that gradually fades out towards the inner part; as a result, the inner zF is largely composed of quiescent cells (Chang et al., 2013). Proliferation arrest coincides with transdifferentiation of zG cells to a zF identity and centripetal migration (Freedman et al., 2013).

Canonical WNT/β-catenin signaling has a prominent position among the molecular pathways that participate in maintaining adrenal cortex homeostasis and zonation (Kim et al., 2008; Leng et al., 2020). Dramatic interventions such as the constitutive activation of β-catenin lead to the expansion of the zG at the expense of the zF and tumor development in aging mice (Berthon et al., 2010; Pignatti et al., 2020). Other mechanisms allow for a more precise fine-tuning of WNT activation levels in the adrenal cortex, such as the negative feedback loop that attenuates WNT signaling based on the activity of the membrane-bound E3 ubiquitin ligase ZNRF3 (Hao et al., 2012). Secreted ligands of the R-spondin (RSPO) family and their cognate receptors LGR4/5/6 form a complex that can bind and remove ZNRF3 from the cell surface, thus potentiating WNT signaling (Nusse and Clevers, 2017; Zebisch et al., 2013). In the mouse adrenal cortex, a diminishing gradient of WNT signaling activity from the zG to the zF is maintained via the localized expression of secreted WNT potentiators, mainly *Wnt4* in the zG, and R-spondins (*Rspo1* and *Rspo3*) in the capsule, and ensures a normal organ size (Basham et al., 2019; Heikkilä et al., 2002; Vidal et al., 2016). Although this knowledge originates mostly from mouse genetic studies, the high prevalence of *CTNNB1* and *ZNRF3* driver mutations in human adrenocortical carcinoma (ACC) shows the relevance of these pathways for human adrenal disease (Assié et al., 2014; Zheng et al., 2016).

The adrenal gland is recognized as one of the most sexually dimorphic non-reproductive organs. For example, many forms of adrenocortical hyperplasia and neoplasia associated with endocrine manifestations, such as Cushing's syndrome, are more frequent among women than men (Lyraki and Schedl, 2021a). This includes benign adrenocortical adenomas (female:male ratio, 4-8:1) (Lacroix et al., 2015; Lindholm et al., 2001) and ACCs (female:male ratio, 1.5-2.5:1) (Ayala-Ramirez et al., 2013; Luton et al., 1990; Scollo et al., 2016). Under normal homeostatic conditions, the mouse adrenal shows a strong dimorphism (Bielohuby et al., 2007; Grabek et al., 2019) and we recently showed that adrenocortical renewal is more rapid in female than in male mice, due to higher activity of cortical AXIN2^+^ progenitors and female-specific recruitment of capsular GLI1^+^ progenitors (Grabek et al., 2019). Sex hormones are implicated in these processes and modulate signaling pathways via yet unknown mechanisms (Dumontet et al., 2018; Grabek et al., 2019).

Even though testicular androgens influence the adrenal cortex, whether this influence is direct and how this influence translates to a reduced susceptibility to hyperplasia is still obscure. To answer these questions, we used a mouse model of disrupted adrenal homeostasis owing to the ectopic expression of *Rspo1* in the adrenal cortex, thus causing moderate WNT signaling hyperactivation. This genetic manipulation results in ectopic proliferation and hyperplasia in female mice, in contrast to cortical thinning and degeneration in males. We show that sexual dimorphism in our model is dependent on testicular androgens, which act directly on adrenocortical cells through their cognate receptor AR (androgen receptor) to cause cell cycle arrest and counteract the mitogenic effect of enhanced WNT signaling.

## RESULTS

### Ectopic expression of *Rspo1* leads to sex-specific adrenocortical hyperplasia or degeneration

**Fig. 1.**
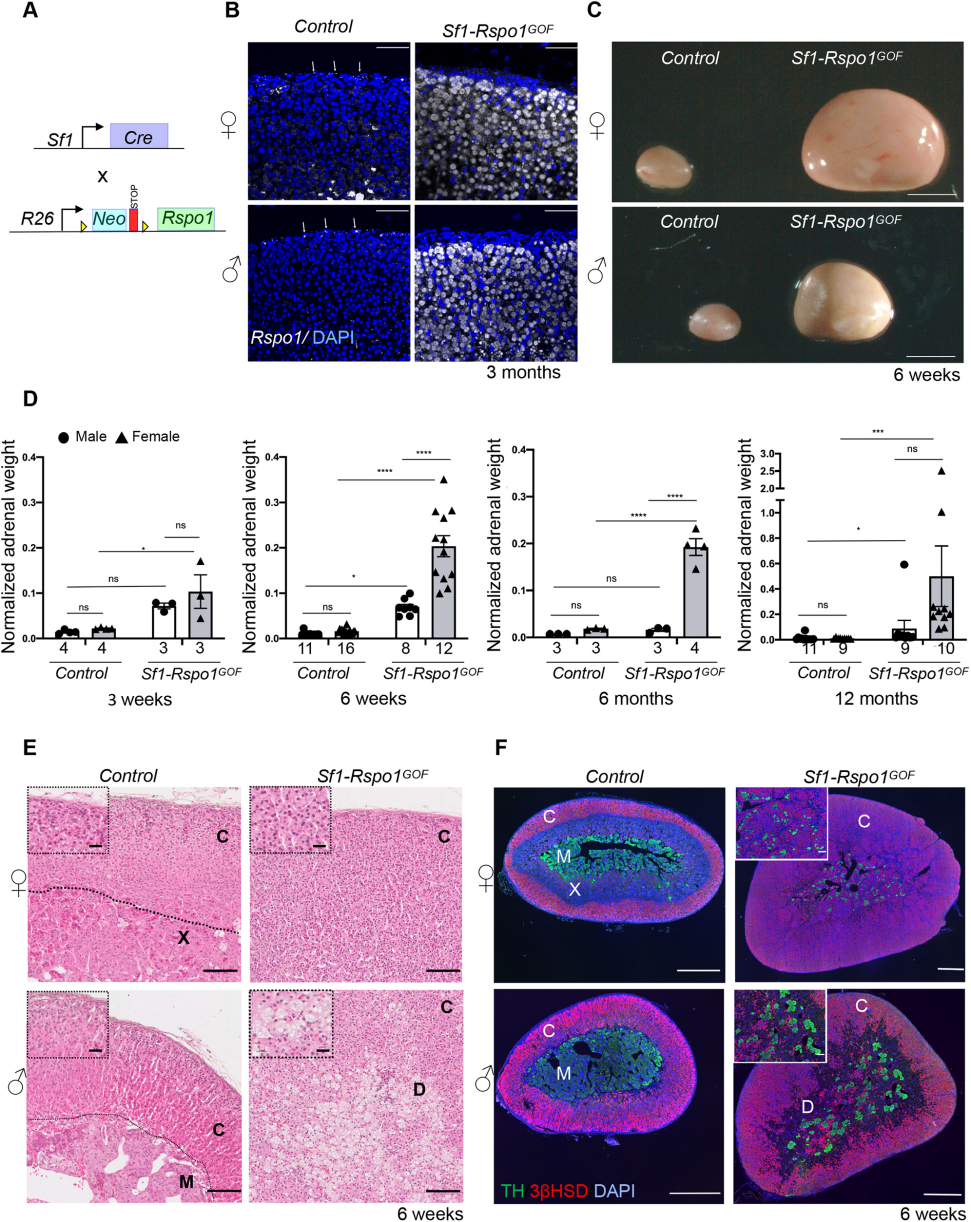
**RSPO1 overexpression leads to sex-specific adrenocortical hyperplasia or degeneration.** (A) Schematic representation of the genetic strategy to overexpress RSPO1 in steroidogenic cells using *Sf1-Cre*. (B) *In situ* hybridization for *Rspo1* using RNA Scope technology on adrenal sections from 3-month-old mice. White arrows mark *Rspo1* mRNA molecules. Scale bars: 50 μm. (C) Representative photographs of adrenal glands from control and *Sf1-Rspo1^GOF^* mice at the end of puberty (6 weeks of age). Scale bars: 1 mm. (D) Graphs of mean adrenal weight normalized to body weight at different ages comparing control mice to *Sf1-Rspo1^GOF^* mice when ectopic RSPO1 expression is driven by *Sf1-Cre* (error bars represent s.e.m.). The numbers below the graph columns represent number of samples in each group (*n*). Statistical analysis for 3 weeks, 6 weeks and 6 months was performed using ordinary two-way ANOVA followed by Tukey's multiple comparisons test. Statistical analysis for 12 months was done using a non-parametric test (Kruskal–Wallis test) owing to the non-Gaussian distribution of the data. Adjusted *P*-values for comparisons at 3 weeks: control females (F) versus *Sf1-Rspo1^GOF^* F, *P*=0.0192; control males (M) versus *Sf1-Rspo1^GOF^* M, *P*=0.1067; *Sf1-Rspo1^GOF^* F versus *Sf1-Rspo1^GOF^* M, *P*=0.5631. Adjusted *P*-values for comparisons at 6 weeks: control F versus *Sf1-Rspo1^GOF^* F, *P*<0.0001; control F versus control M, *P*=0.0239; *Sf1-Rspo1^GOF^* F versus *Sf1-Rspo1^GOF^* M, *P*<0.0001; control M versus *Sf1-Rspo1^GOF^* M, *P*=0.0184. Adjusted *P*-values for comparisons at 6 months: control F versus *Sf1-Rspo1^GOF^* F, *P*<0.0001; control M versus *Sf1-Rspo1^GOF^* M, *P*=0.9500; *Sf1-Rspo1^GOF^* F versus *Sf1-Rspo1^GOF^* M, *P*<0.0001. Adjusted *P*-values for comparisons at 12 months: control F versus *Sf1-Rspo1^GOF^* F, *P*=0.0008; control M versus *Sf1-Rspo1^GOF^* M, *P*=0.0144; *Sf1-Rspo1^GOF^* F versus *Sf1-Rspo1^GOF^* M, *P*=0.4853. ns, not significant; *P<0.05; ***P<0.001; ****P<0.0001. (E) H&E staining of adrenals from 6-week-old mice. Scale bars: 100 μm (for insets, 20 μm). (F) Immunofluorescence staining for tyrosine hydroxylase (TH, marker of the adrenal medulla) and 3βHSD (marker of steroidogenic cells), using adrenal sections from 6-week-old mice. Note that the size of the scale bar is different for each picture. Scale bar: 500 μm (for insets, 100 μm). C, cortex; X, X-zone; M, medulla; D, degeneration.

In order to generate a mouse model for adrenocortical hyperplasia, we sought to disrupt the gradient of canonical WNT signaling activation by ectopically expressing R-spondin 1 (RSPO1) in the adrenal cortex. We used a Cre-inducible *Rspo1* gain-of-function (GOF) allele (De Cian et al., 2017; Rocha et al., 2015) and the *Sf1-Cre* transgene that drives Cre recombinase expression in SF1^+^ tissues including the adrenal cortex (Bingham et al., 2006) (*Sf1-Rspo1^GOF^* mice) (Fig. 1A). In control adult animals, *Rspo1* expression was restricted to the outer adrenal capsule, in agreement with previous research (Vidal et al., 2016). By contrast, *Sf1-Rspo1^GOF^* mice showed expression throughout the adrenal cortex (Fig. 1B) and dramatically increased total mRNA levels regardless of sex (Fig. S1A).

Ectopic *Rspo1* expression in our models resulted in striking hyperplasia of the adrenal glands in 6-week-old mice (Fig. 1C). Because the *Sf1-Cre* line drives expression of the GOF allele already during embryogenesis, hyperplasia was noticeable in pre-pubertal pups and was comparable between male and female mice at 3 weeks of age (Fig. 1D). After puberty, however, the phenotype evolved in a highly sexually dimorphic manner. Whereas female adrenals from *Sf1-Rspo1^GOF^* mice further increased in size, male adrenals remained smaller, and their size even regressed as they aged (Fig. 1D; 6 weeks, 6 months and 12 months). Ordinary two-way ANOVA confirmed the interactive effect of sex and *Rspo1* overexpression on adrenal weight at 6 weeks and 6 months (*P*<0.0001) but not at 3 weeks (*P*=0.46). Thus, puberty appears to be a critical period for the development of sexual dimorphism in our model.

Next, we conducted a histological analysis to examine the cellular composition of *Sf1-Rspo1^GOF^* adrenals. According to Hematoxylin and Eosin (H&E) analysis at 6 weeks of age, all the female GOF adrenals analyzed exhibited diffuse atypical hyperplasia in the cortex (*n*=5) (Fig. 1E). The inner cortex was composed of steroidogenic cells expressing 3βHSD (encoded by *Hsd3b*), although its expression seemed reduced compared to control adrenals, whereas the medulla was fragmented (Fig. 1F). Male GOF adrenals, however, displayed non-neoplastic degenerative changes in the form of markedly vacuolated, polynucleated cells. At 6 weeks, all the male *Sf1-Rspo1^GOF^* adrenals (*n*=6) exhibited these degenerative changes to a varying degree (Fig. 1E), which were negative for the steroidogenic marker 3βHSD (Fig. 1F). By 3 months of age, degenerative areas expanded significantly in all the male *Sf1-Rspo1^GOF^* adrenals (*n*=3), leading to cortical thinning (Fig. S1B). These degenerative changes affected female adrenals to a much lesser degree (2/3 adrenals examined at 3 months displayed only a few abnormal cells) (Fig. S1B). zG expansion is a known consequence of the constitutive activation of β-catenin in the adrenal cortex (Berthon et al., 2010; Pignatti et al., 2020). However, in *Sf1-Rspo1^GOF^* adrenals, the zG was not expanded in either sex, as shown by immunostaining with the zG markers DAB2 and LEF1 (Fig. S1C,D).

To assess whether ectopic expression of *Rspo1* affected the endocrine activity of the hyperplastic adrenals, we measured plasma steroids of control and GOF animals at 6 weeks of age by liquid chromatography coupled with tandem mass spectrometry (LC-MS/MS). Levels of the mineralocorticoid hormone aldosterone and its precursor 18-OH-corticosterone were not significantly changed among our experimental groups. Levels of the glucocorticoid corticosterone and another steroid hormone precursor, 11-deoxycorticosterone, showed a mild decrease in female and increase in male *Sf1-Rspo1^GOF^* mice, although none of these changes reached statistical significance (Fig. S2). Analysis of the adrenocorticotropic hormone-responsive differentiation marker AKR1B7 (Sahut-Barnola et al., 2000) showed loss of expression in subpopulations of cells of the inner cortex, which explains the absence of hormone overproduction despite the striking hyperplasia (Fig. S1E). Plasma levels of the androgens testosterone and dihydrotestosterone (DHT) did not differ significantly among control and *Sf1-Rspo1^GOF^* mice (Fig. S2).

As the *Sf1-Rspo1^GOF^* animals aged, the diffuse hyperplasia and cortical thinning gave way to increased frequency of well-circumcised benign nodules and adenomas (Fig. S3) (6/8 *Sf1-Rspo1^GOF^* males and 6/8 *Sf1-Rspo1^GOF^* females of 12 months). Importantly, 1/8 aging *Sf1-Rspo1^GOF^* females had a well-differentiated adrenal carcinoma with capsular invasion (Fig. S3). None of the controls or male *Sf1-Rspo1^GOF^* animals displayed any malignant tumor; thus, sexual dimorphism might also characterize tumor progression, but analysis of more animals is required to draw this conclusion.

Taken together, ectopic *Rspo1* expression exerts a highly sexually dimorphic effect on the adrenal cortex. In female mice, it leads to diffuse hyperplasia of the adrenal cortex without increased endocrine activity. On the contrary, male GOF adrenals display expansive degenerative lesions and cortical thinning, although compensating activity of the remaining cortex ensures that insufficiency of steroid hormones is avoided.

### Ectopic expression of *Rspo1* leads to female-specific ectopic proliferation

**Fig. 2.**
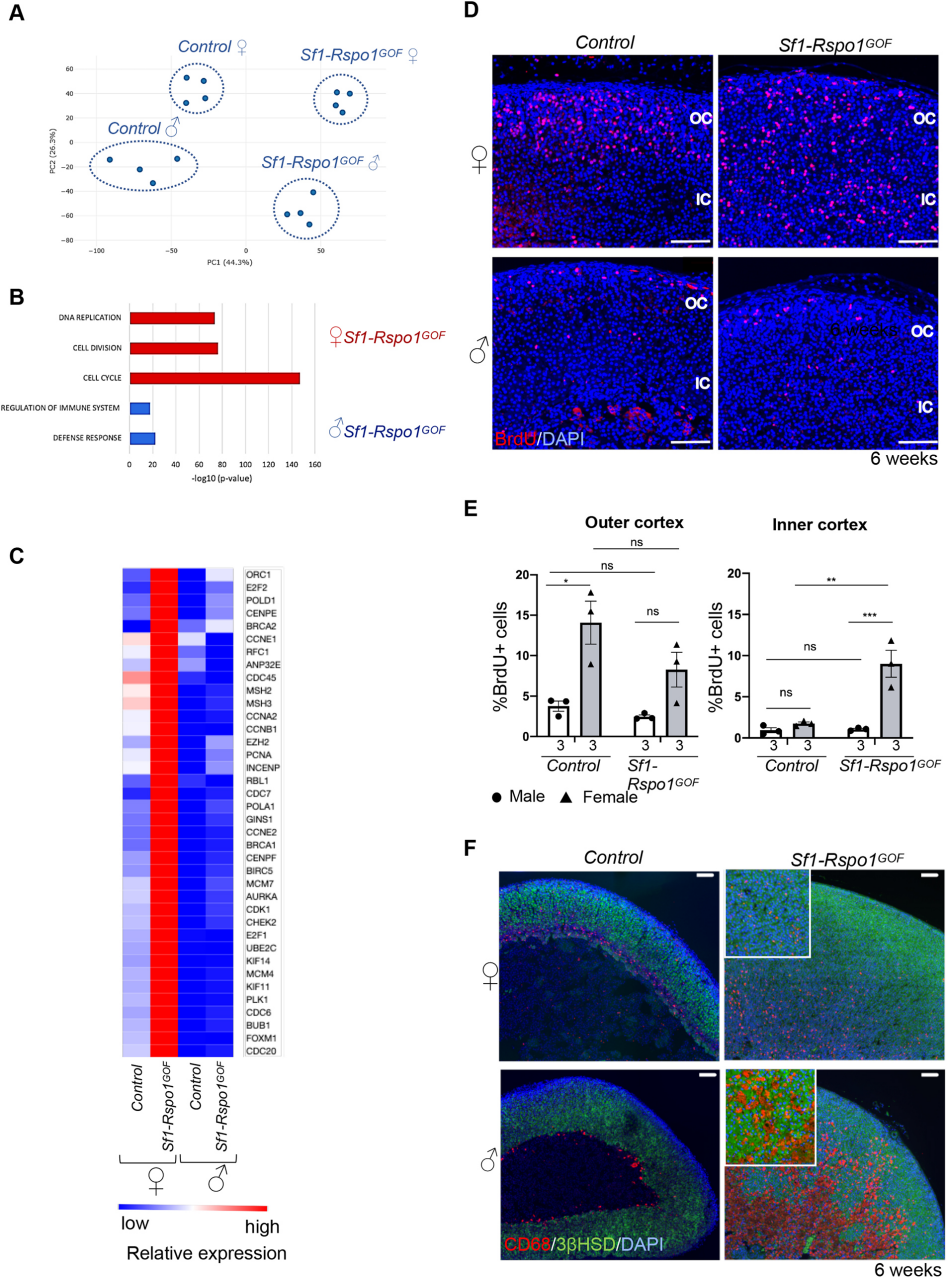
**Transcriptomic analysis of *Sf1-Rspo1^GOF^* adrenals during puberty reveals sex-specific regulation of cell cycle and immune responses.** (A) Principal component analysis (PCA) plot of gene expression data in control and *Sf1-Rspo1^GOF^* male versus female adrenals during puberty (4 weeks of age). Each dot corresponds to an independent biological replicate. (B) Top enriched Gene Ontology terms in the clusters representing genes that are highly expressed in male or female *Sf1-Rspo1^GOF^* adrenals compared to those from the other experimental groups. *P*-value indicates statistical significance. (C) Heatmap representation of relative differences in expression of proliferation-related genes among experimental groups. Relative expression among the groups is indicated by the color code. The expression of all genes shown here is known to be induced during either the G1/S or the G2/M transitions of the cell cycle. (D) Representative immunofluorescence images for BrdU. Scale bars: 100 μm. OC, outer cortex; IC, inner cortex. (E) BrdU proliferation analysis shown as mean percentage of proliferating cells over total number of cells in the adrenal cortex of 6-week-old mice (error bars represent s.e.m.). The numbers below the graph columns represent number of samples in each group (*n*=3). The area close to the capsule (outer cortex) is distinguished from the deeper layers (inner cortex). Statistical analysis was performed using ordinary two-way ANOVA followed by Tukey's multiple comparisons test. Adjusted *P*-values for the outer cortex graph: control females (F) versus *Sf1-Rspo1^GOF^* F, *P*=0.1623; control F versus control males (M), *P*=0.0128; *Sf1-Rspo1^GOF^* F versus *Sf1-Rspo1^GOF^* M, *P*=0.1638; control M versus *Sf1-Rspo1^GOF^* M, *P*=0.9506. Adjusted *P*-values for the inner cortex graph: control F versus *Sf1-Rspo1^GOF^* F, *P*=0.0013; control F versus control M, *P*=0.8967; *Sf1-Rspo1^GOF^* F versus *Sf1-Rspo1^GOF^* M, *P*=0.0007; control M versus *Sf1-Rspo1^GOF^* M, *P*=0.9996. ns, not significant; *P<0.05; **P<0.01; ***P<0.001. (F) Immunofluorescence staining for CD68 (marker of murine macrophages) and 3βHSD (marker of steroidogenic cells) using adrenal sections from 6-week-old mice. Scale bars: 100 µm.

The striking sexual dimorphism in our *Sf1-Rspo1^GOF^* model is reminiscent of the female bias observed in human adrenal diseases. To further investigate its molecular underpinnings, we conducted mRNA sequencing and differential expression analysis of whole adrenals from control and *Sf1-Rspo1^GOF^* mice of both sexes during puberty (at 4 weeks), a timepoint before the occurrence of male-specific degeneration of adrenals (Fig. S4A) [data available at NCBI's Gene Expression Omnibus (Barrett et al., 2013) with the accession number GSE178958]. As expected, principal component analysis (PCA) identified the presence of the *Rspo1^GOF^* allele as a major component in our RNA sequencing (RNA-seq) experiment. Strikingly, sex was responsible for 26.3% of the variation in gene expression patterns among our experimental groups (Fig. 2A). To gain insights into the molecular changes occurring in different subgroups, we next performed gene set enrichment analysis (GSEA) using the Molecular Signature Database (Broad Institute, Cambridge, MA, USA). Genes enriched in *Sf1-Rspo1^GOF^* animals of both sexes compared to controls (corresponding to cluster 1 of the heatmap in Fig. S4B) were related to β-catenin upregulation (Fevr et al., 2007; Sansom et al., 2007) or targets of the β-catenin-associated transcription factor LEF1 (Xie et al., 2005), a finding consistent with an hyperactivation of canonical WNT signaling by *Rspo1*. Genes specifically upregulated in female *Rspo1^GOF^* adrenals (corresponding to cluster 3 of the heatmap in Fig. S4B) were found to be primarily related to cell cycle regulation, DNA replication and repair, and cell division (Fig. 2B). A more in-depth GSEA revealed that targets of transcription factors belonging to the E2F family and the DREAM complex (Fischer et al., 2016), critical repressors of cell cycle genes participating in the G1/S and G2/M transitions, were specifically upregulated in female *Sf1-Rspo1^GOF^* adrenals (Fig. 2C). On the contrary, genes highly expressed in male *Sf1-Rspo1^GOF^* adrenals (corresponding to cluster 2 of the heatmap in Fig. S4B) were associated with the regulation of the immune system and defense response (Fig. 2B). Moreover, an unbiased comparison of differentially regulated genes between male and female *Sf1-Rspo1^GOF^* adrenals via GSEA confirmed that ‘DNA replication’ and ‘immune response’ were among the top enriched pathways in female and male *Sf1-Rspo1^GOF^* adrenals, respectively (Fig. S4C). Of note, GSEA revealed a downregulation of catecholamine secretion in *Sf1-Rspo1^GOF^* adrenals compared to controls, probably reflecting the fragmentation of the medulla that we observed in our histological analysis (Fig. 1F).

To confirm the conclusions drawn from the mRNA sequencing experiment, we analyzed DNA replication in the adrenal cortex at 6 weeks of age via bromodeoxyuridine (BrdU) incorporation (Fig. 2D,E). No differences in proliferation between control and GOF adrenals were observed in the outer cortex, which makes up the proliferating zone under wild-type circumstances. However, proliferation was dramatically increased in the inner cortex of *Sf1-Rspo1^GOF^* females, which mostly consists of quiescent cells in the control animals. Surprisingly, no increase was observed in male *Sf1-Rspo1^GOF^* adrenals. Ordinary two-way ANOVA analysis confirmed the interactive effect of sex and *Rspo1* overexpression in regulating proliferation in the inner cortex (*P*=0.0028). Sex-specific proliferation was not observed before puberty, as male and female GOF adrenals both showed increased proliferation rates during embryogenesis (Fig. S5A,B), whereas ordinary two-way ANOVA analysis showed the absence of interactive effect between sex and the presence of the transgene at this early stage (*P*=0.8311).

Previous work from our group has shown the dominant role of RSPO3 over RSPO1 in the mouse adrenal cortex. Even though *Rspo3* deletion led to cortical atrophy both during development and in adulthood, *Rspo1* deletion did not produce an apparent defect (Vidal et al., 2016). In order to test whether the phenotype we observed was specific to *Rspo1^GOF^*, we analyzed the effect of ectopic *Rspo3* expression in the adrenal cortex, taking advantage of a previously published *Rspo3^GOF^* allele in the *Rosa26* locus (Hilkens et al., 2017) and the *Sf1-Cre* system (Fig. S6A). *Rspo3* overexpression phenocopied our *Sf1-Rspo1^GOF^* model in terms of sex-specific hyperplasia and ectopic proliferation at 6 weeks of age (Fig. S6B,C). However, we were not able to compare the two models directly because the expression of the *Rspo3* transgene was not uniform (Fig. S6D). Thus, we conclude that overexpression of either R-spondin affects the adrenal cortex in the same manner.

### Male *Rspo1^GOF^* adrenals display abnormal macrophage accumulation

Because our transcriptomic analysis revealed an enrichment for immune-related genes among the cluster of genes specifically upregulated in male *Rspo1^GOF^* adrenals, we examined the expression of macrophage markers by immunofluorescence staining. CD68 represents a marker of inflammation abundantly expressed in macrophages and is also detected in other cell types of the myeloid lineage (Chistiakov et al., 2017). CD68 is expressed in wild-type adrenals at 6 weeks of age at the border between the cortex and the medulla or the X-zone (Fig. 2F). By contrast, CD68 marked small cells scattered around the cortex in the hyperplastic *Sf1-Rspo1^GOF^* female adrenals of the same age, whereas in male *Sf1-Rspo1^GOF^* adrenals, CD68 marked cells increasing in size that adopted a ‘foamy’ morphology, fused and contributed to the formation of the degenerative lesions (Fig. 2F). Several genes expressed in macrophages and monocytes, as well as pan-immune cell markers, were upregulated in male *Sf1-Rspo1^GOF^* adrenals (Fig. S7A). Moreover, IBA1 (*Aif1*), a macrophage marker that has been shown to be induced in *Star* knockout animals (Ishii et al., 2012), was strongly expressed in male *Sf1-Rspo1^GOF^* adrenals, but not in their female counterparts (Fig. S7B). On the contrary, male-specific foamy cell formation was not observed when *Rspo1* overexpression was activated by the adrenal cortex-specific aldosterone synthase (AS) *Cyp11b2*-*Cre* line (Freedman et al., 2013) (more details about this genetic model are provided in the ‘AR signaling determines sexual dimorphism in Rspo1GOF adrenals’ section) (Fig. S7C). The discrepancy can be explained by the slower rate of recombination of *Cyp11b2-Cre* (5 weeks of age for the whole adrenal cortex to express the transgene) compared to that of *Sf1-Cre* (the transgene is expressed already during embryonic life). Therefore, our results indicate that the sex-specific degeneration has its origins in early, possibly embryonic, dysregulation. Overall, our data suggest that male *Sf1-Rspo1^GOF^* adrenals develop a sex-specific inflammatory profile, characterized by increased presence of monocytes and macrophages, that culminates in cortical thinning with increased age.

### Sex-specific pattern of canonical WNT signaling activation in *Rspo1^GOF^* adrenals

**Fig. 3.**
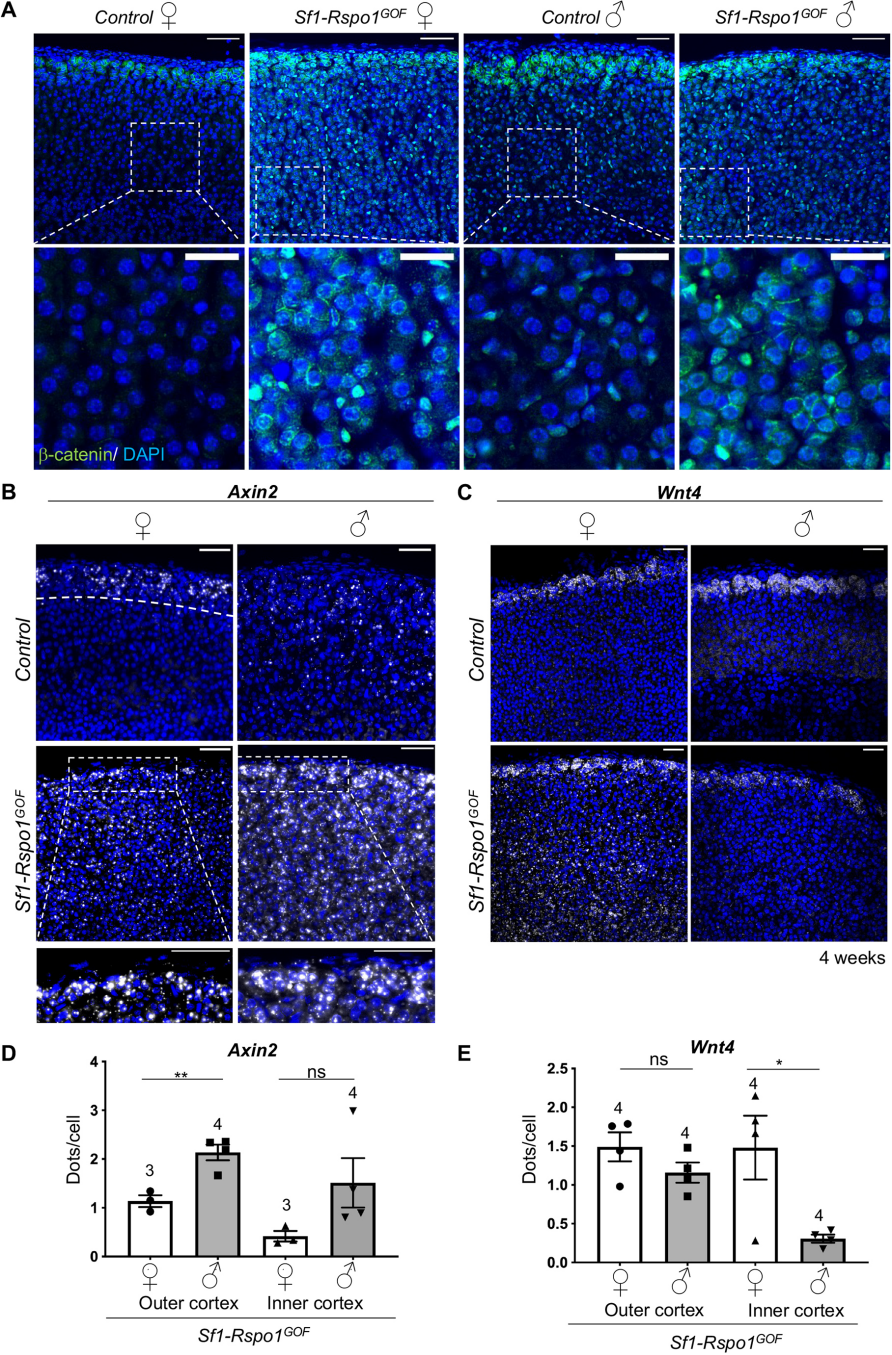
**RSPO1 overexpression induces the expansion of canonical WNT activity zone in the adrenal cortex in a sexually dimorphic manner.** (A) Immunofluorescence staining for β-catenin using adrenal sections from 4-week-old mice. Scale bar: 50 μm (top panels); 20 μm (bottom panels). (B,C) *In situ* hybridization using the RNA Scope technology for *Axin2* (B), a target of canonical WNT signaling, and *Wnt4* (C) at 4 weeks of age. Insets represent high-power images of the outer cortex in GOF animals. The outer cortex is denoted by a dotted line in B (female control; top left panel). Scale bars: 50 μm. (D,E) Graphs showing the mean number of *Axin2* (D) and *Wnt4* (E) transcripts per cell in the outer cortex or inner cortex, based on RNA Scope-based detection on adrenal sections from 4-week-old mice (error bars represent s.e.m., whereas each dot represents an independent biological replicate). The numbers above the graph columns represent number of samples in each group (*n*). Statistical analysis was performed using unpaired two-tailed *t*-test. *P*-value comparing male versus female outer cortex for *Axin2*=0.0058. *P*-value comparing male versus female inner cortex=0.1302. *P*-value comparing male versus female outer cortex for *Wnt4*=0.1963. *P*-value comparing male versus female inner cortex=0.0301. ns, not significant; *P<0.05; **P<0.01.

In order to identify the cause of sexual dimorphism in our model, we tested whether sex influences canonical WNT signaling activation. To focus on primary events, we chose an early timepoint (4 weeks) when sex-specific differences in proliferation start to emerge, but degeneration is not yet obvious in male adrenals. In agreement with previous research (Leng et al., 2020), β-catenin shows strong membrane immunoreactivity in the zG but is absent in the inner cortex of control animals (apart from cells containing small spindle-like nuclei that do not have the characteristic morphology of steroidogenic cells). However, in *Sf1-Rspo1^GOF^* adrenals, we observed increased membrane, nuclear and perinuclear β-catenin immunoreactivity in the inner cortex, in addition to its characteristic zG pattern (Fig. 3A). To quantify this disruption of the characteristic WNT signaling gradient, we performed RNA Scope *in situ* hybridization (ISH) analysis for *Axin2* and *Wnt4*, two markers of canonical WNT signaling activation. Although *Axin2* is a well-characterized target of canonical WNT signaling (Jho et al., 2002), *Wnt4* is thought to be a driver of canonical WNT signaling in the adrenal cortex (Basham et al., 2019; Vidal et al., 2016). Consistent with previous data (Basham et al., 2019), the expression of these two genes follows a gradient of diminishing expression from the outer to the inner cortex in control animals, which was disrupted in our GOF model (Fig. 3B,C). Interestingly, the expression of these two genes became sexually dimorphic in *Sf1-Rspo1^GOF^* adrenals, with *Axin2* expression being increased in males (reaching statistical significance in the outer cortex) and *Wnt4* expression being increased in females (reaching statistical significance in the inner cortex) (Fig. 3D,E).

### AR signaling determines sexual dimorphism in *Rspo1^GOF^* adrenals

**Fig. 4.**
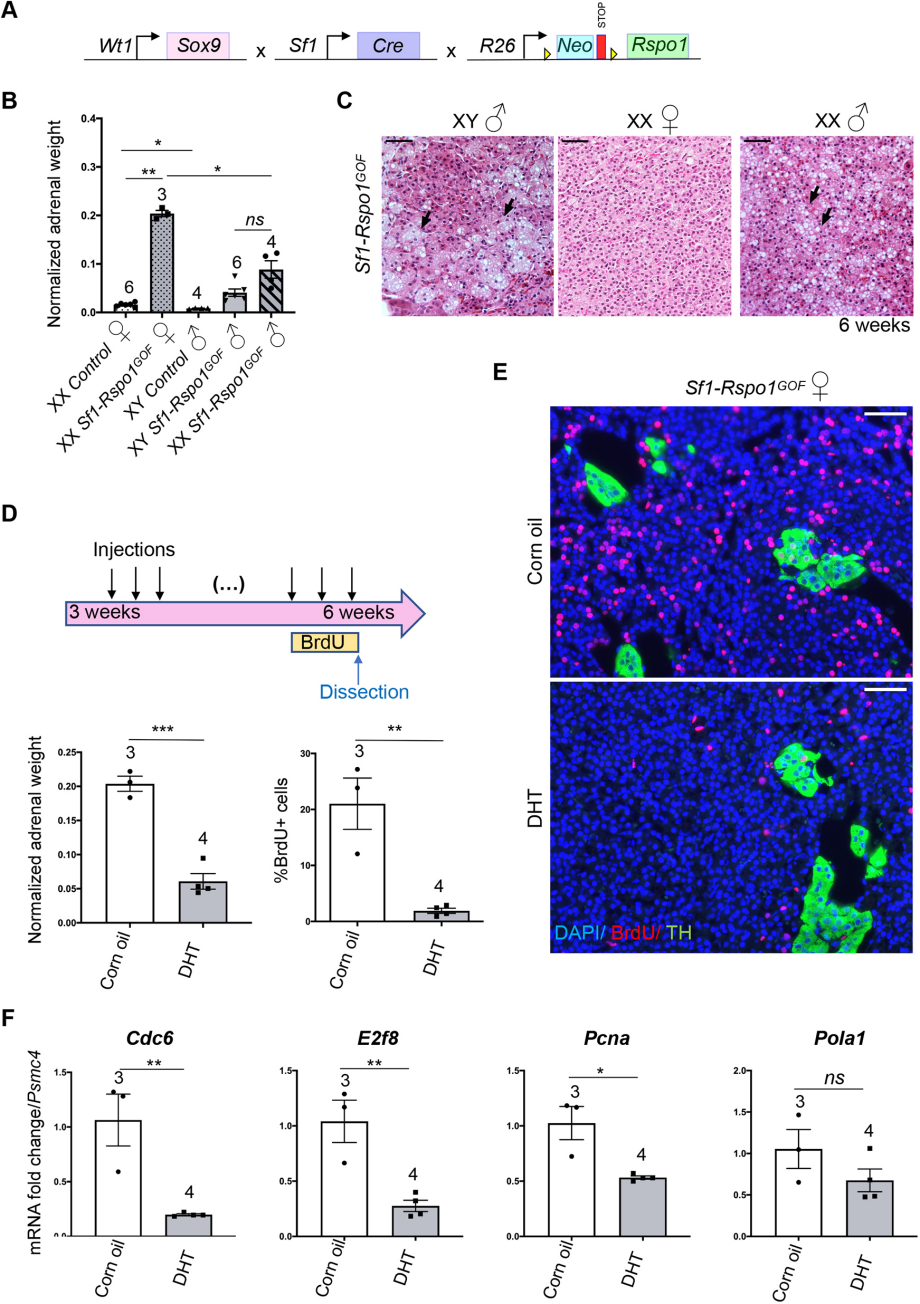
**Sexually dimorphic response to RSPO1 overexpression is caused by androgens.** (A) Schematic representation of the genetic strategy to combine RSPO1 overexpression in steroidogenic tissues with female-to-male sex-reversal caused by ectopic SOX9 expression under control of a *Wt1* regulatory sequence. (B) Mean adrenal weight normalized to whole-body weight at 6 weeks. ‘XX’ and ‘XY’ denote sex chromosomes and ‘XX ♂’ indicates sex-reversed *Tg(Wt1-Sox9)^Tg/0^* mice. Statistical analysis was performed with one-way Welch's ANOVA followed by Dunnett's T3 multiple comparisons test. Adjusted *P*-values: XX control F versus XX *Sf1-Rspo1^GOF^* F, *P*=0.0048; XX control F versus XY control M, *P*=0.0367; XX *Sf1-Rspo1^GOF^* F versus XX *Sf1-Rspo1^GOF^* M, *P*=0.0231. (C) H&E staining on adrenal sections from 6-week-old XY and XX *Sf1-Rspo1^GOF^* male mice. Black arrows point to vacuolated cells that form degenerative lesions. Scale bars: 50 μm. (D) Female *Sf1-Rspo1^GOF^* mice were treated with DHT or corn oil daily for 3 weeks during puberty. Graphs represent mean adrenal weight normalized to total body weight (bottom left; *P*=0.0003) and percentage of proliferating cells (BrdU^+^) in the adrenal cortex (bottom right; *P*=0.0043). Statistical analysis was conducted using unpaired two-tailed *t*-test. (E) Representative immunofluorescence images for BrdU and TH (marker of the medulla). Scale bars: 50 μm. (F) RT-qPCR analysis of the expression of genes related to the G1/S cell cycle transition. Graphs represent mean fold-change in expression comparing corn oil to DHT treated adrenals (normalized to *Psmc4* expression). Statistical analysis was conducted with unpaired *t*-test. *P*-values: 0.1964 (*Pola1*), 0.0066 (*E2f8*), 0.0073 (*Cdc6*) and 0.0117 (*Pcna*). DHT: dihydrotestosterone. All error bars represent s.e.m. The numbers above the graph columns represent number of samples in each group (*n*). ns, not significant; *P<0.05; **P<0.01; ***P<0.001.

To explain sexual dimorphism in adrenocortical hyperplasia, it is essential to dissect the role of sex hormones versus the role of sex chromosomes. We took advantage of a sex reversal model in which constitutive ectopic expression of a *Wt1-Sox9* gene in XX gonads leads to the development of testes (Vidal et al., 2001) (Fig. 4A). Sex-reversed *Sf1-Rspo1^GOF^* males [*Gt(Rosa)26Sor^cCAG-Rspo1^*^/+^; *Sf1-cre^Tg/0^*; *Wt1-Sox9^Tg/0^* XX] displayed significantly lower normalized adrenal weight than *Sf1-Rspo1^GOF^* females [*Gt(Rosa)26Sor^cCAG-Rspo1/+^*; *Sf1-cre^Tg/0^* XX] (Fig. 4B) and developed vacuolated cells in the inner cortex at 6 weeks of age (Fig. 4C), thus phenocopying XY *Sf1-Rspo1^GOF^* male adrenals. These data indicate that gonadal rather than chromosomal sex is responsible for the sexual dimorphism.

We have shown previously that androgens can suppress progenitor proliferation during normal adrenal cortex homeostasis (Grabek et al., 2019). To test whether androgens would also modify female-specific hyperplasia in our mouse model, we chose to treat *Sf1-Rspo1^GOF^* female mice daily with the androgen DHT during puberty (3-6 weeks of age) (Fig. 4D). Expression analysis revealed a slight reduction of the AR gene (*Ar*), as well as a dramatic increase in the expression of *Susd3*, a putative androgen-responsive gene in the adrenal cortex (as suggested by our RNA-seq data and previous transcriptomic analyses; El Wakil et al., 2013) (Fig. S8B). Moreover, DHT treatment dramatically reduced normalized adrenal weight and proliferation levels in the adrenal cortex (Fig. 4D,E) but did not lead to histological changes such as cytoplasmic vacuolization (Fig. S8A). The reduction of proliferation levels in DHT-treated animals was accompanied by a reduction in expression levels of genes associated with the G1/S transition of the cell cycle (*E2f8*, *Pcna*, *Cdc6* and *Pola1*) (Fischer and Müller, 2017) (Fig. 4F). However, the expression of cell cycle genes that are also known β-catenin targets (*Ccnd1* and *Myc*) (Shtutman et al., 1999; Van de Wetering et al., 2002) did not show statistically significant changes after treatment, suggesting that the effect of the androgens on cell cycle progression is not due to direct regulation of β-catenin targets (Fig. S8B). Furthermore, DHT treatment led to an increase in *Axin2* expression levels and to a decrease in *Wnt4* expression levels (Fig. S8B), similarly to what RNA Scope ISH analysis suggested regarding the effect of sex on the expression of WNT-related genes (Fig. 3C,E). Interestingly, long-term DHT treatment did not cause consistent changes in the subcellular localization of β-catenin in the inner cortex (Fig. S8C).

**Fig. 5.**
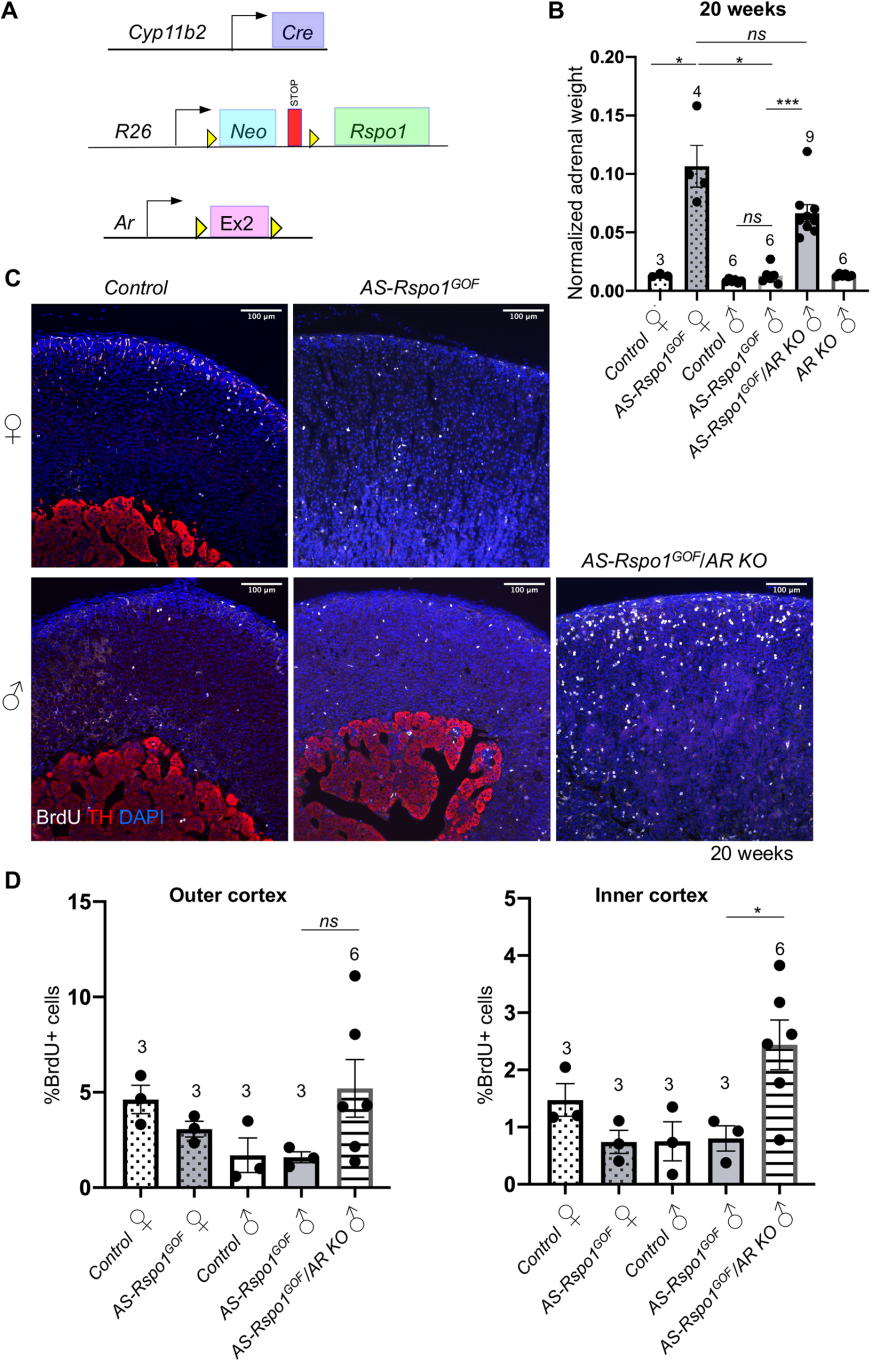
**Androgen receptor deletion in male *AS-Rspo1^GOF^* adrenocortical cells abolishes sexual dimorphism in the development of hyperplasia.** (A) Schematic representation of the genetic strategy to knock-out *Ar* and simultaneously overexpress RSPO1 in adrenocortical cells using the *Cyp11b2-Cre* driver. (B) Adrenal weight normalized to body weight at 20 weeks. Statistical analysis was performed using Welch's one-way ANOVA followed by Dunnett's T3 post hoc test. Adjusted *P*-values: control F versus *AS-Rspo1^GOF^* F, *P*=0.0454; control M versus *AS-Rspo1^GOF^* M, *P*=0.5777; *AS-Rspo1^GOF^* F versus *AS-Rspo1^GOF^* M, *P*=0.0468; *AS-Rspo1^GOF^* F versus *AS-Rspo1^GOF^*/*Ar KO* M, *P*=0.3370; *AS-Rspo1^GOF^* M versus *AS-Rspo1^GOF^*/*Ar KO* M, *P*=0.0001. (C) Representative immunofluorescence images for tyrosine hydroxylase (TH) and BrdU, indicating proliferating cells. Scale bars: 100 μm. (D) BrdU proliferation analysis shown as a percentage of proliferating cells over the total number of cells in the adrenal cortex of 20-week-old mice. The area close to the capsule (outer cortex) is distinguished from the deeper layers (inner cortex). Unpaired two-tailed *t*-test was employed to compare values for *AS-Rspo1^GOF^* male and *AS-Rspo1^GOF^*/*Ar KO* male. Adjusted *P*-values: 0.1478 (outer cortex) and 0.0410 (inner cortex). All error bars represent s.e.m. The numbers above the graph columns represent number of samples in each group (*n*). ns, not significant; *P<0.05; ***P<0.001.

**Fig. 6.**
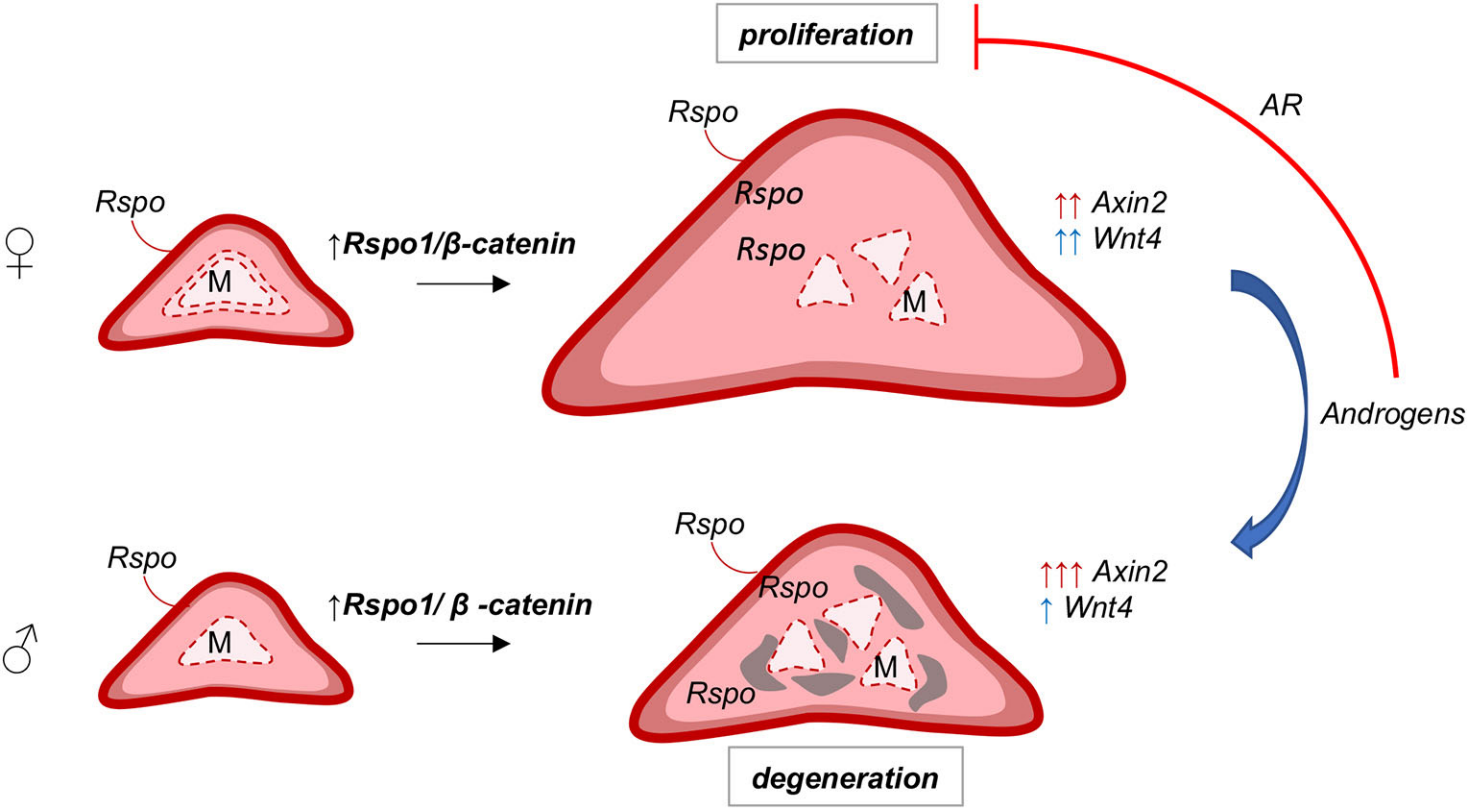
**Sex-specific effects of *Rspo1* overexpression in the adrenal cortex.** Localized expression of R-spondins (by the capsule) maintains high levels of WNT/β-catenin signaling and proliferation in the outer cortex. Ectopic expression of *Rspo1* by steroidogenic cells leads to increased WNT/β-catenin signaling in the inner cortex, as indicated by ectopic expression of *Wnt4* and *Axin2*, and ectopic proliferation that causes adrenocortical hyperplasia and fragmentation of the medulla (M). On the contrary, in male adrenals or in female adrenals treated with androgens (DHT), ectopic *Rspo1* causes differential effects on *Wnt4* and *Axin2* expression, whereas ectopic proliferation is prevented via activation of AR signaling.

Adrenal cortex homeostasis depends on complex endocrine interactions with the pituitary-hypothalamic axis and the gonads (Goel et al., 2014). Having established a role for DHT in suppressing hyperplasia, we asked whether this effect is direct or involves feedback loops via other organs. To answer this question, we took advantage of a conditional knockout (KO) allele for *Ar* (De Gendt et al., 2004) that we activated simultaneously with the *Rspo1* transgene. To exclude extra-adrenal effects, we employed the adrenal cortex-specific AS *Cyp11b2*-*Cre* line (Freedman et al., 2013) (Fig. 5A) that becomes activated in the zG at birth and – owing to the centripetal displacement of cortical cells – leads to recombination throughout the cortex by approximately 5 weeks of age. Given the slower recombination rate compared to that of *Sf1-Cre*, we analyzed adrenals at 20 weeks of age. Similarly to *Sf1-Cre*, *Cyp11b2-Cre* activation of *Rspo1(AS-Rspo1^GOF^)* resulted in female adrenal hyperplasia, whereas growth of the male adrenals remained comparable to that of wild-type controls (Fig. 5B). Strikingly, simultaneous deletion of the *Ar* allele caused a significant increase of male adrenals to a weight comparable to that found in female counterparts (Fig. 5B). Moreover, male *AS-Rspo1^GOF^*/*Ar KO* and female *AS-Rspo1^GOF^* adrenals appeared similar on the histological level, characterized by cortical hyperplasia (Fig. S9A). BrdU-based analysis confirmed an increase in proliferation in male *Rspo1^GOF^/Ar KO* adrenals, particularly in the inner cortex. The observed variability among different animals (Fig. 5C,D) is likely due to incomplete deletion of the *Ar* allele in clusters of adrenocortical cells (Fig. S9B). Surprisingly, *Ar* deletion led to a mild increase of β-catenin accumulation in cells in certain areas of the inner cortex of *AS-Rspo1^GOF^* male adrenals (Fig. S9C). However, quantification of *Axin2* expression in the outer and inner cortex using RNA Scope ISH (Fig. S9D) revealed no significant changes, suggesting that *Ar* deletion does not translate into a change of transcriptional activity of β-catenin. Taken together, our results demonstrate that androgens act directly on adrenocortical cells by engaging their cognate receptor and cause proliferation arrest, contributing to a differential susceptibility to adrenocortical hyperplasia among the sexes (Fig. 6).

## DISCUSSION

Canonical WNT/β-catenin signaling gradients determine a plethora of processes, ranging from embryonic development to the maintenance of adult stem cell niches, whereas their dysregulation is linked to carcinogenesis in humans (Nusse and Clevers, 2017). High β-catenin levels in the zG of the mouse adrenal cortex, supported by secreted R-spondins, are paramount to maintaining regular renewal and zonation (Kim et al., 2008; Vidal et al., 2016). Here, we report adrenal hyperplasia of the zF owing to R-spondin overexpression and mild WNT signaling activation. Although our model phenotypically resembles the effects of *Znrf3* deletion described before (Basham et al., 2019), we also describe a striking, androgen-dependent sexual dimorphism in phenotypic development, which was not reported in this earlier study. Notably, the two studies corroborate the tied role of R-spondin ligands and the ZNRF3 ubiquitin ligase in maintaining the WNT/β-catenin signaling gradient and localized proliferation in the adrenal cortex.

We found that immune system activation and abnormal proliferation, two major pillars of tumorigenesis, are regulated in a sex hormone-specific manner in *Rspo1^GOF^* mice. Indeed, males displayed the recruitment of macrophages, monocytes and dendritic cells when *Sf1-Cre* was used to induce *Rspo1* overexpression (*Sf1-Rspo1^GOF^*). On the histological level, this recruitment manifested as foamy cell formation, cytoplasmic vacuolization and tissue degeneration. Remarkably, we did not observe the same male-specific foamy macrophage formation in the *AS-Rspo1^GOF^* model. This discrepancy might reflect the differential rates of Cre activation, as *Sf1-Cre* is active during embryogenesis, whereas the *AS-Cre* line results in cortex-wide recombination only at 5[Supplementary-material sup1]weeks of age (Freedman et al., 2013). We can hypothesize that embryonically derived macrophages are involved, such as the CX3CR1^+^ population, recently demonstrated by a study analyzing sex-specific differences in adrenal cortex macrophage distribution and expression profile (Dolfi et al., 2022). Alternatively, macrophage recruitment could result from extra-adrenal *Rspo1* overexpression, as *Sf1-Cre* is also expressed in other endocrine organs, such as the gonads. We consider this scenario less likely, as plasma levels of androgens do not differ significantly between control and *Sf1-Rspo1^GOF^* animals, thus excluding the hypothesis that excess of gonadal androgens induces macrophage recruitment in males.

Recently, an independent study reported a male-specific, proinflammatory environment and abundant recruitment of macrophages in the *Znrf3* knockout adrenal cortex in response to the appearance of senescent cortical cells. The authors suggest that androgen-dependent macrophage recruitment may be a significant contributor to female bias in ACC (Wilmouth et al., 2022). We did not observe histological changes consistent with macrophage recruitment after DHT treatment, but this may be due to the relatively short timeframe of androgen treatment in our study. Of note, the finding of macrophage infiltration upon *Rspo1* overexpression and *Znrf3* deletion is contrary to studies in other systems in which WNT/β-catenin activation has been reported to favor exclusion of infiltrating immune cells from the tumor microenvironment (Muto et al., 2023). Although macrophage recruitment is likely to contribute to the degenerative phenotype in male mice, androgen-induced proliferation arrest appears to be cell intrinsic and independent of macrophage activity. Indeed, deletion of *Ar* induces hyperproliferation in our *AS-Rspo1^GOF^* mice, a model that does not show macrophage recruitment.

However, a high proliferation index is an important feature of ACC, associated with malignant rather than benign tumors and worse prognosis (Crona and Beuschlein, 2019). Moreover, dysregulation of WNT/β-catenin signaling and cell cycle regulation [via the p53/retinoblastoma-associated protein (RB1) axis] are significant hallmarks of ACC and the pathways most frequently affected by driver mutations (Assié et al., 2014; Zheng et al., 2016). In this context, our findings can contribute to a greater understanding of sex bias in ACC frequency and the development of personalized therapies. In future studies, it will be important to test whether androgen administration in the context of other ACC models (Borges et al., 2020) also suppresses proliferation and tumor growth. Of note, it has been described that DHT treatment of human ACC cells leads to growth arrest (Rossi et al., 1998).

Androgens are potent suppressors of the hypothalamic-pituitary-adrenal axis at the level of the hypothalamus and the pituitary gland, which in turn regulate corticosterone production by the adrenal cortex (Seale et al., 2004). Distinguishing direct versus indirect effects of sex hormones in gonadectomy and DHT-treatment experiments has therefore been difficult. We show here that removing the cognate DHT receptor from adrenocortical cells (*Ar* deletion) renders male adrenals susceptible to hyperplasia and hyperproliferation and abolishes sexual dimorphism. Thus, this is the first report of AR signaling directly suppressing proliferation in the adrenal cortex. Of note, it has been shown before that AR signaling influences adrenal weight and X-zone regression (Gannon et al., 2019).

Although the genetic evidence for suppression of steroidogenic cell proliferation by AR is striking, the molecular mechanisms underlying this process are less clear. Direct antagonism between AR and WNT/β-catenin signaling has been suggested before for epidermal stem cells (Kretzschmar et al., 2015) and prostate cancer cells (Mulholland et al., 2003). In our model, we did not find evidence for a sex-specific global reduction of β-catenin signaling in male compared to female adrenals. On the contrary, *Axin2* expression – a recognized marker of WNT/β-catenin signaling – was reproducibly increased in the inner cortex of males compared to females and was induced following DHT treatment. This observation agrees with the work from Dumontet et al. (2018), which suggested that androgens positively affect WNT signaling in the adrenal cortex, thus counteracting PKA signaling and cortical cell turnover. This molecular action is believed to contribute to the female susceptibility to Cushing’s syndrome. However, whether AR is directly involved in this phenomenon is questionable, as we did not find markedly altered *Axin2* expression following *Ar* deletion in the male GOF adrenal cortex.

Another possibility is that AR suppresses transcription of cell cycle genes independently of WNT signaling. Indeed, although we found that the expression of several G1/S cell cycle transition genes was repressed by DHT treatment, these known targets of β-catenin remained almost stable. Interestingly, it has already been reported that AR acts as a transcriptional repressor for a subset of DNA replication genes by recruiting RB1 to their promoters (Gao et al., 2016).

The finding that AR suppresses proliferation in the adrenal cortex might seem surprising, given the well-characterized association of AR with prostate growth, benign prostate hyperplasia and prostate tumorigenesis (Dai et al., 2017). However, even in prostate cancer, AR can have opposing effects on cell cycle regulation, depending on ligand concentration, duration of treatment and the extent of GOF alterations in AR signaling in the context of malignant transformation (Chatterjee et al., 2019; Gao et al., 2016; Litvinov et al., 2006). Moreover, it has been suggested that AR acts as a tumor suppressor in the context of breast cancer (Hickey et al., 2021). Our study contributes to understanding of AR signaling complexity and tissue specificity and highlights one prominent cause of sexual dimorphism in a non-reproductive organ. Further work will be required to delineate the precise mechanistic underpinnings of androgen-specific suppression of proliferation in the context of the adrenal cortex.

## MATERIALS AND METHODS

### Animal husbandry and genetics

All animal work was conducted according to national and international guidelines and approved by the local ethical committee [Comité Institutionnel d'Éthique Pour l'Animal de Laboratoire (CIEPAL): APAFIS#6001-201606281711255 v6, APAFIS#14137-2018030216239792 v1] and the French Ministry of Agriculture. The mouse strains (*Mus musculus*) used in this study have been reported previously: *Rspo1^GOF^* (De Cian et al., 2017; Rocha et al., 2015), *Sf1-Cre* (Bingham et al., 2006), *Wt1-Sox9* (Vidal et al., 2001), *Rspo3^GOF^* (Hilkens et al., 2017), *Cyp11b2-Cre* (Freedman et al., 2013) and *Ar^flox^* (De Gendt et al., 2004). Mice heterozygous for the *Rspo1^GOF^* allele and the *Sf1-Cre* allele are referred to as ‘*Sf1-Rspo1^GOF^*’ [*Gt(Rosa)26Sor^cCAG-Rspo1/+^*; *Sf1-Cre*^Tg/0^], whereas mice with genotypes that do not permit the expression of the GOF allele are referred to as controls [*Gt(Rosa)26Sor^+/+^*; *Sf1-Cre*^Tg/0^, *Gt(Rosa)26Sor^+/+^*; *Sf1-Cre*^0/0^ or *Gt(Rosa)26Sor^cCAG-Rspo1/+^*; *Sf1-Cre*^0/0^]. The expression of a *Wt1-Sox9* allele distinguishes sex-reversed *Sf1-Rspo1^GOF^* males [*Gt(Rosa)26Sor^cCAG-Rspo1/+^*; *Sf1-Cre*^Tg/0^; *Wt1-Sox9*^Tg/0^ XX] from *Sf1-Rspo1^GOF^* females [*Gt(Rosa)26Sor^cCAG-Rspo1/+^*; *Sf1-Cre*^Tg/0^; *Wt1-Sox9*^0/0^ XX] and males [*Gt(Rosa)26Sor^cCAG-Rspo1/+^*; *Sf1-Cre*^Tg/0^; *Wt1-Sox9*^0/0^ XY]. Mice heterozygous for the *Rspo1^GOF^* allele and *Cyp11b2* (aldosterone synthase)-Cre are referred to as ‘*AS-Rspo1*^*GOF*^' [*Gt(Rosa)26Sor*^*cCAG-Rspo1/+*^; *Cyp11b2*^*Cre/+*^], compared to the genotype that does not permit expression of the GOF allele, referred to as control [*Gt(Rosa)26Sor*^*+/+*^; *Cyp11b2*^*+/+*^]. When the gene encoding for AR is deleted in males, mice are referred to as ‘*AS-Rspo1*^*GOF*^*/Ar KO*' [*Gt(Rosa)26Sor*^*cCAG-Rspo1/+*^; *Cyp11b2*^*Cre/+*^; *Ar*^*flox/Y*^]. Mouse lines were maintained on a mixed genetic background. Both males and females were analyzed at various ages as indicated in the main text, whereas littermates were preferentially compared.

### Immunofluorescence and histology

For immunofluorescence and H&E analysis of paraffin-embedded samples, mouse left adrenal tissues were fixed overnight at 4°C with 4% paraformaldehyde, progressively dehydrated and paraffin embedded. For H&E staining, sections of 5 mm were rehydrated and stained with Eosin and Mayer's Hematoxylin (3 min each), before being dehydrated again and mounted using an anhydrous mounting medium. Carcinoma and adenoma formation was verified by expert pathologists. For immunofluorescence, 5 mm sections were unmasked with PT Link (Dako Agilent Pathology Solutions) at pH 6 or 9. Sections were blocked for 1 h with 10% normal donkey serum (Jackson Immunoresearch), 3% bovine serum albumin (BSA; Sigma-Aldrich) and 0.1% Tween-20 (Sigma-Aldrich). Primary antibodies were applied overnight at 4°C diluted in 3% normal donkey serum, 3% BSA and 0.1% Tween-20 as explained in Table S2. The next day, sections were incubated at room temperature with secondary antibodies donkey anti-rabbit Alexa Fluor 647, donkey anti-goat Alexa Fluor 555, donkey anti-mouse Alexa Fluor 555, donkey anti-rabbit Alexa Fluor 555 and donkey anti-mouse Alexa Fluor 647 diluted 1:400 in PBS (see Table S2). Sections were then mounted in antifade mounting medium with DAPI (Vectashield). The Mouse-on-Mouse immunodetection kit (Vector Laboratories) was used to improve the signal for mouse primary antibodies according to the manufacturer's instructions. For anti-AR and anti-IBA1 immunohistochemistry, sections were incubated with biotinylated donkey anti-rabbit secondary antibodies (see Table S2) and the signal was revealed using the Vectastain Elite ABC-HRP peroxidase kit and the ImmPACT NovaRED HRP substrate (Vector Laboratories). Counterstaining was performed using 50% Harris Hematoxylin for 30 s, followed by incubation in 0.1% sodium bicarbonate solution for 1 min at room temperature. The antibodies used were all commercially available with reported validation profiles. Appropriate negative controls (without the primary antibody) were used in all immunostaining experiments.

### Proliferation quantification

For proliferation quantification in the adrenal cortex, 1 mg/ml of BrdU (Sigma-Aldrich) was dissolved in autoclaved water with 2% sugar and given to mice as drinking water for 3 days. Anti-BrdU immunofluorescence was conducted as described above. Quantification was performed using the HALO image analysis platform (Indica Labs) on whole-section mosaic images obtained with the Vectra Polaris imaging system (Akoya Biosciences), excluding the medulla based on tyrosine hydroxylase (TH) staining. The number of BrdU-positive cells was expressed as the percentage of the total number of cells in each zone based on DAPI staining. When the distinction between ‘outer’ and ‘inner’ cortex is made, we refer to the zone <80 mm from the capsule and >80 mm from the capsule, respectively. At least three biological replicates (individual mice) and three non-consecutive sections for each biological replicate were analyzed.

### RNA scope ISH

Adrenal sections were fixed with 4% paraformaldehyde overnight at room temperature and paraffin embedded. Fresh 5 μm sections were subjected to single-molecule ISH using the RNA Scope 2.5 High Definition-Red assay (ACD Biotechne), according to the manufacturer's instructions. Images were acquired with a Zeiss apotome upright microscope or a Zeiss LSM NLO 780 confocal microscope. For quantification purposes, whole-section mosaic images were acquired with the Vectra Polaris imaging system (Akoya Biosciences) and quantified using the FISH Multiplex v1.1 module of the HALO image analysis platform (Indica Labs). Outer cortex represents the proliferative zone and includes the zG and 12±1% of the zF for male control animals, and 25±7% of the zF for female control animals (the zF in the females is considered shorter due to the presence of the X-zone). The inner cortex includes the rest of the zF up to the X-zone (if present) or the medulla.

### Gene expression analysis

RNA was extracted from mouse right adrenals using the RNeasy mini kit (QIAGEN) according to the manufacturer's instructions. cDNA synthesis was performed using M-MLV reverse transcriptase (Invitrogen) and random primers. The obtained cDNA was used as template in a real-time quantitative PCR (RT-qPCR) reaction using the SYBR Green Master Mix (Roche) and a LightCycler 1.5 (Roche) or a QuantStudio 5 thermocycler (Applied Biosystems). Expression levels were normalized to the housekeeping gene *Psmc4*, analyzed using the 2^-ΔΔCt^ method and presented as fold-change values compared to a reference sample. The primers used are listed in Table S1.

### Hormonal treatment and analysis of hormone levels in plasma

*Sf1-Rspo1^GOF^* females were injected twice daily subcutaneously with 5a-androstan-17b-ol-3-one (Sigma-Aldrich) (37.5 μg in 5% ethanol and corn oil) or oil only from 3-6 weeks of age, when they were sacrificed. For the measurement of adrenal steroids in mouse plasma, 6-week-old animals were sacrificed in the morning and core trunk blood was collected in tubes containing 5 μl of 0.5 M EDTA. The samples were centrifuged for 5 min at 4000 ***g*** at 4°C to separate the plasma, which was promptly frozen at −80°C until analysis. Steroid hormones were quantified by LC-MS/MS as described previously (Peitzsch et al., 2015).

### RNA-seq and analysis

RNA was extracted from mouse right adrenals using the RNeasy mini kit (QIAGEN) according to the manufacturer's instructions. Four biological replicates were analyzed for each group (control male, control female, *Sf1-Rspo1^GOF^* male and *Sf1-Rspo1^GOF^* female). The sample quality was assessed using a Bioanalyzer 2100 (Agilent Technologies) and a RNA integrity number (RIN) cut-off value of 7.0 was applied.

Library preparation and sequencing, as well as differential expression analysis were conducted by Novogene. Briefly, library preparation was conducted using a NEBNext Ultra RNA Library Prep kit for Illumina (New England Biolabs). After cluster generation, the library preparations were sequenced on an Illumina platform and 125/150 bp paired-end reads were generated. Raw data (raw reads) of FASTQ format were firstly processed through in-house Perl scripts. In this step, clean data (clean reads) were obtained by removing reads containing adapter, reads containing poly-*N* and low-quality reads from raw data. All the downstream analyses were based on the clean data with high quality. The annotation was performed based on the GRCm38 genome assembly and downloaded from Ensembl. The index of the reference genome was built using Bowtie v2.2.3 and paired-end clean reads were aligned to the reference genome using TopHat v2.0.12. To count the reads numbers mapped to each gene, HTSeq v0.6.1 was used. The fragments per kilobase of transcript per million mapped reads (FPKM) value of each gene was calculated based on the length of the gene and read count mapped to this gene. Differential expression analysis was performed using the DESeq R package (v1.18.0). The resulting *P*-values were adjusted using the Benjamini and Hochberg approach for controlling the false discovery rate (FDR). Genes with an adjusted *P*-value <0.05 found by DESeq were assigned as differentially expressed.

For downstream analyses, PCA plot and heatmaps were designed using the Phantasus website tools (Kleverov et al., 2022 preprint), using log_10_(FPKM+1) expression values from differentially expressed genes as templates. Enriched gene sets were calculated using the Molecular Signatures database with an FDR q-value threshold of 0.05. GSEA (Subramanian et al., 2005) was conducted after DESeq2 was used via the GenePattern platform (Reich et al., 2006) to calculate differentially expressed genes between male and female GOF adrenals.

### Statistical analysis

Statistical analysis was conducted as indicated in each figure legend using the GraphPad Prism 7 software. Given the complex nature of our genetic models, the number of samples were limited, and the sample size (*n*) reflects the number of animals available for each genotype.

## Supplementary Material

10.1242/dmm.050053_sup1 Supplementary information
