# Supplementary material for: Crosstalk between androgen receptor and WNT/β-catenin signaling causes sex-specific adrenocortical hyperplasia in mice
Source: Dis Model Mech. 2023 May 10;16(6):dmm050053. doi: 10.1242/dmm.050053 (PMC10184674; doi:10.1242/dmm.050053)
Supplement: Supplementary information [file dmm-16-050053-s1.pdf]

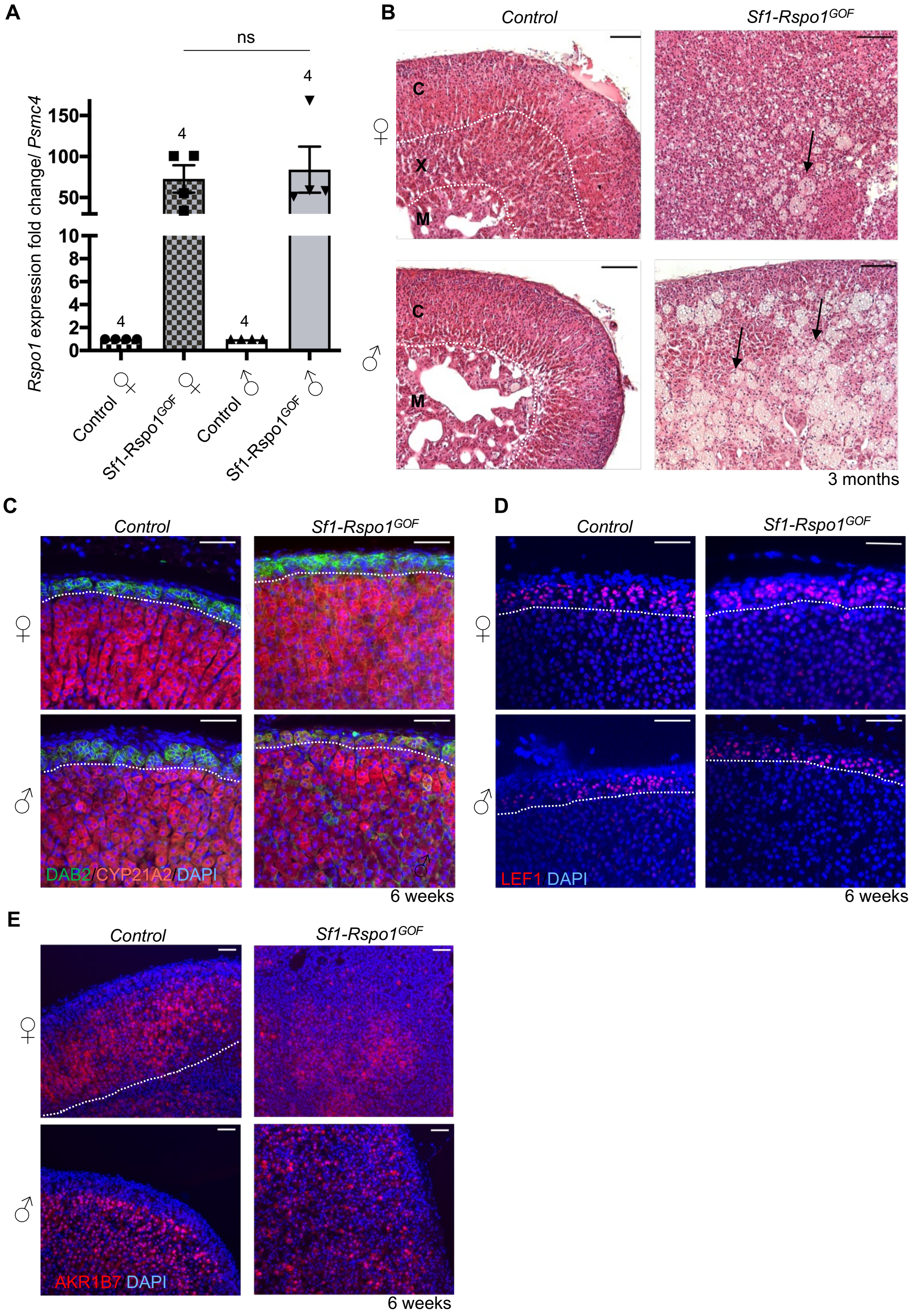

Disease Models & Mechanisms • Supplementary information

Fig. S1. Characterization of RSPO1 overexpression and its effects on adrenocortical cells.
A) RT-qPCR analysis of *Rspo1* expression relative to *Psmc4* in adrenals from 6-week-old mice, shown as mean fold change in *Sf1-Rspo1*^{GOF} adrenals compared to sex-matched controls. The numbers below the graph columns represent number of samples in each group (n). The numbers above the graph columns represent number of samples in each group (n=4). Statistical analysis was performed using unpaired two-tailed t-test (P=0.7442). Error bars represent the standard error of the mean (SEM). B) H&E staining of adrenal sections from 3-month-old mice. Black arrows point to the vacuolated cells forming degenerative lesions. C: cortex, X: x-zone, M: medulla. Scale bar: 100 µm. C) Immunofluorescence staining for DAB2 (marker of the zG) and CYP21A2 (marker of adrenal steroidogenic cells), using adrenal sections from 6-week-old mice. Scale bar: 50 µm. D) Immunofluorescence staining for LEF1 (marker of the zG) on adrenal sections from 6-week-old mice. Scale bar: 50 µm. E) Immunofluorescence staining for the aldo-keto reductase AKR1B7 using adrenal sections from 6-week-old mice. Scale bar: 50 µm.

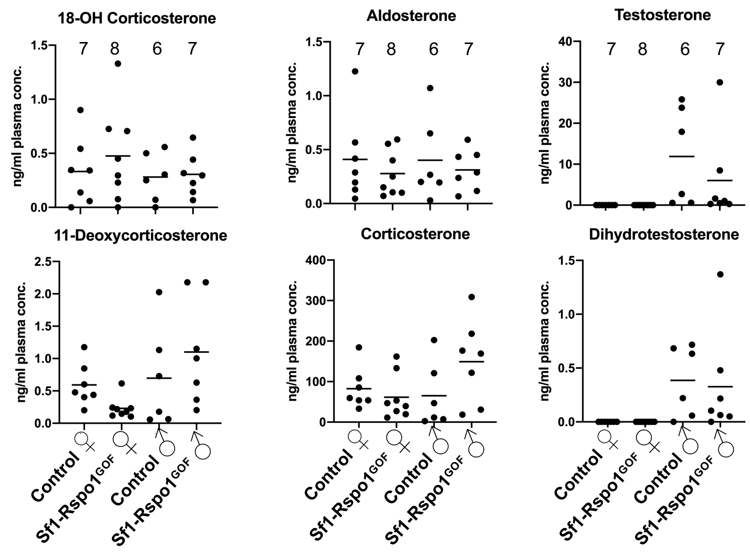

Fig. S2. Plasma steroid levels. Quantification of adrenal and testicular steroids in the plasma of control and *Sf1-Rspo1*^{GOF} mice at 6 weeks of age by liquid chromatography paired with tandem mass spectrometry (LC-MS/MS). The numbers above the graph columns represent number of samples in each group (n).

Disease Models & Mechanisms • Supplementary information

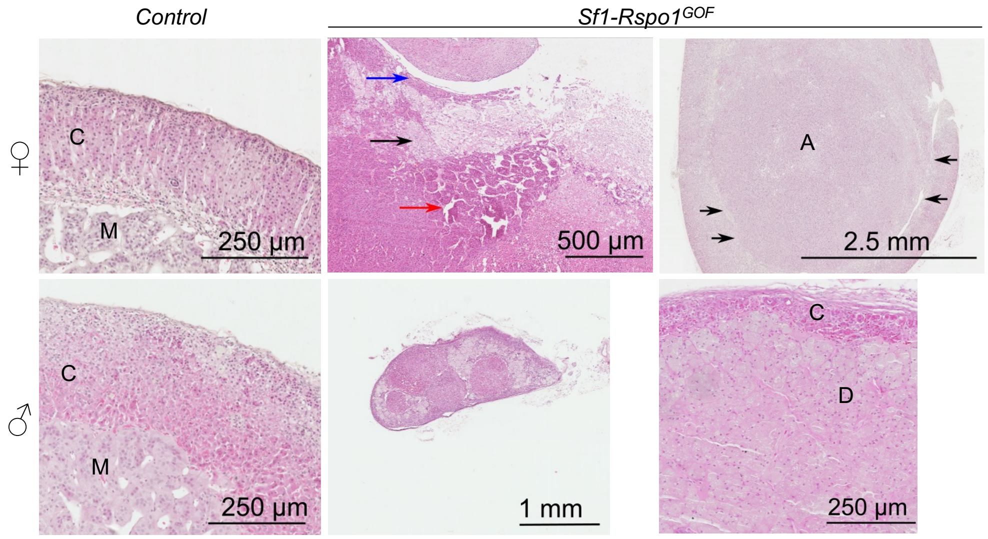

Fig. S3. Ectopic RSPO1 expression leads to the development of nodules and neoplasia in aging animals. On the top panel, representative H&E images of 12-month-old female control and *Sf1-Rspo1*^{GOF} adrenals are shown. Both female *Sf1-Rspo1*^{GOF} adrenals have tumors: in the middle, the adrenal is completely effaced by an adrenocortical carcinoma. Capsular invasion (blue arrow) and areas of extended necrosis (black arrow) and nest-like cell organization (red arrow) can be seen. On the left, the adrenal harbors a well-circumcised adrenocortical adenoma. On the bottom panel, representative H&E staining on 12-month-old male control and *Sf1-Rspo1*^{GOF} adrenals shows the extensive degeneration and cortical thinning paired with incidences of nodular hyperplasia in the *Sf1-Rspo1*^{GOF} adrenals. C: cortex, M: medulla, D: degeneration, A: adenoma.

Disease Models & Mechanisms • Supplementary information

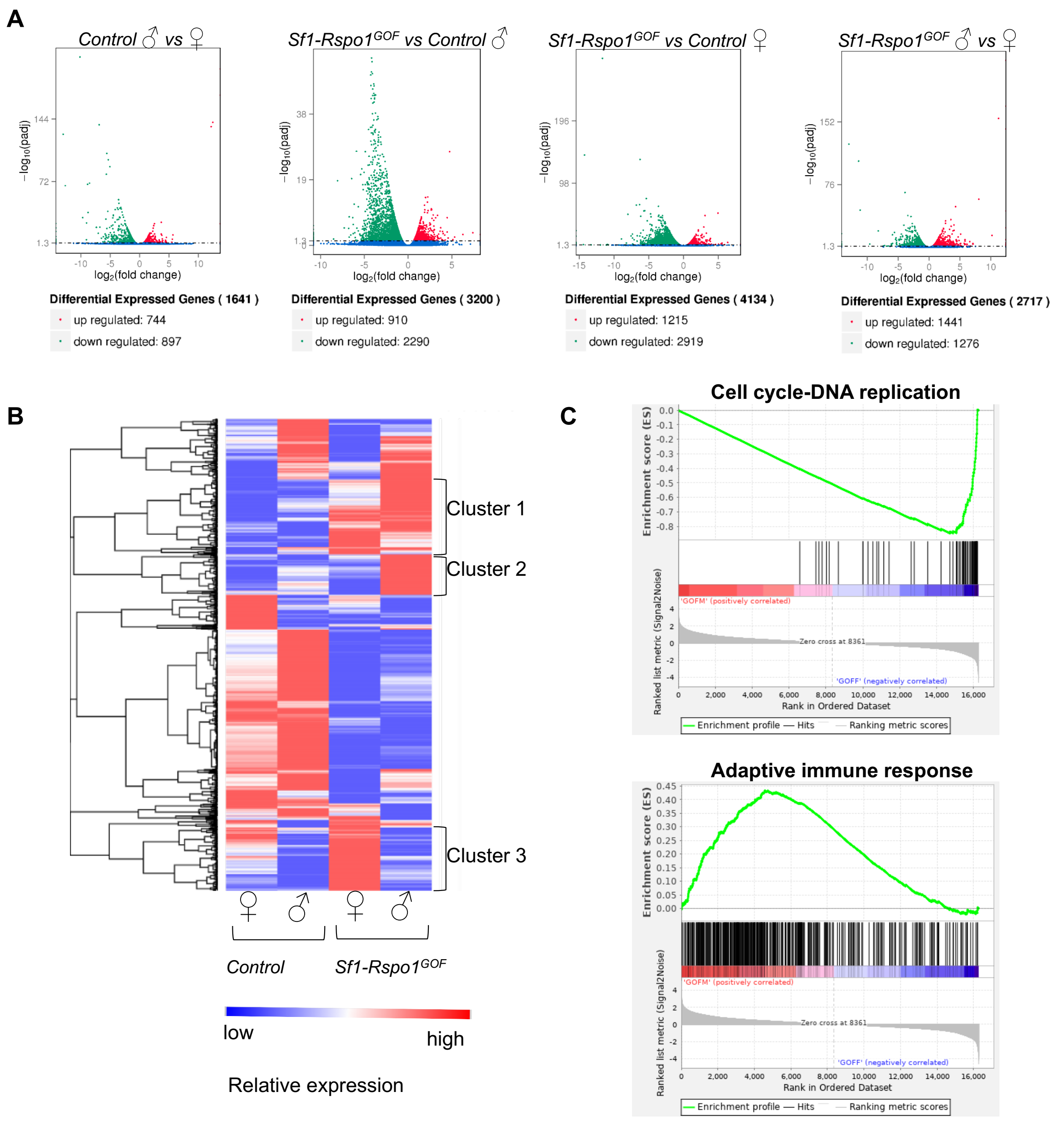

Fig. S4. Analysis of differential gene expression between groups. A) Volcano plots differentially expressed genes between pairs of experimental groups. B) Hierarchical clustering and heatmap representation of differential gene expression among control and *Sf1-Rspo1*^{GOF}, male and female adrenals. Relative expression differences are shown in color code. C) Enrichment plots for gene ontology (GO) terms produced by GSEA analysis comparing male ('GOFM') to female ('GOFF') *Sf1-Rspo1*^{GOF} adrenals.

Disease Models & Mechanisms • Supplementary information

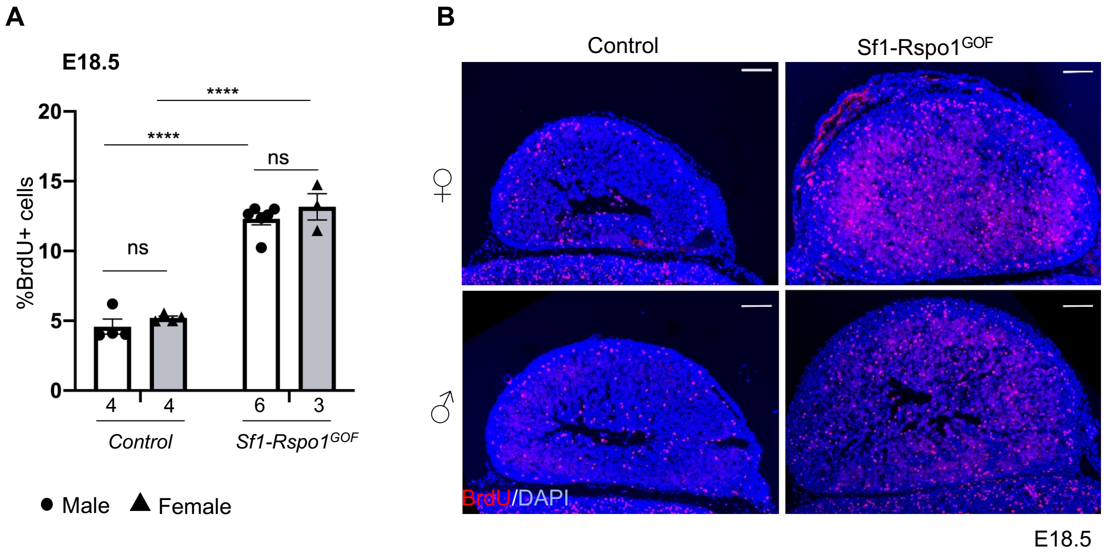

Fig. S5. Male and female *Rspo1*^{GOF} embryos display equal rates of adrenocortical cell proliferation. A) BrdU proliferation analysis shown as mean percentage of proliferating cells over total number of cells in the adrenal cortex of E18.5 mouse embryos (error bars represent SEM). The numbers below the graph columns represent number of samples in each group (n). Statistical analysis was conducted using ordinary two-way ANOVA followed by Tukey's multiple comparison's test. Adjusted p-values: Control F vs *Sf1-Rspo1*^{GOF} F: P<0.0001, Control M vs *Sf1-Rspo1*^{GOF} M: P<0.0001, *Sf1-Rspo1*^{GOF} F vs *Sf1-Rspo1*^{GOF} M: P=0.6680. B) Representative images of BrdU immunostaining in E18.5 adrenals. Scale bars: 100 µm.

Disease Models & Mechanisms • Supplementary information

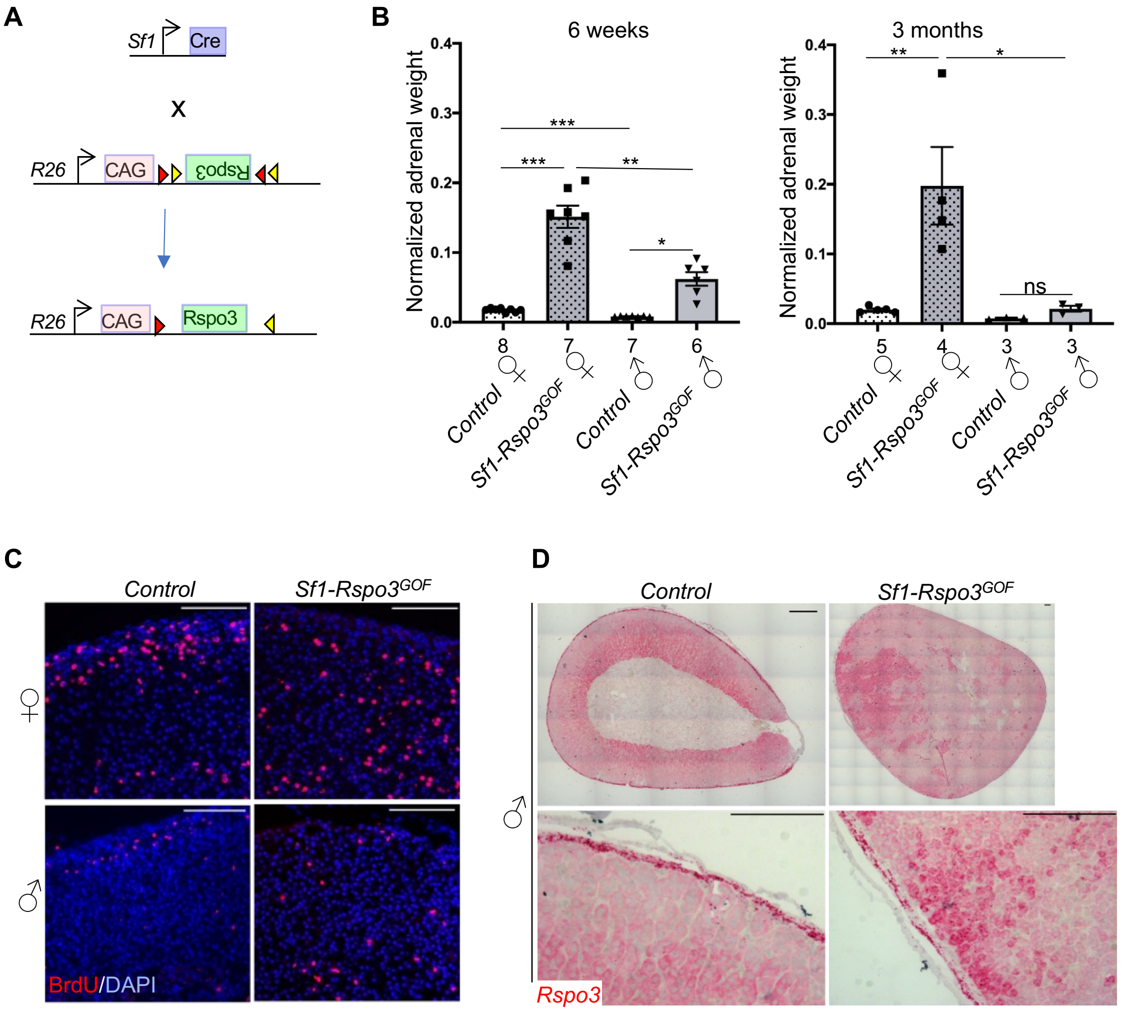
A
Sf1
Cre
X
R26
CAG
Rspo3
R26
CAG
Rspo3
B
6 weeks
3 months
Normalized adrenal weight
0.4
0.3
0.2
0.1
0.0
***
***
**
*
**
*
ns
8
7
7
6
5
4
3
3
Control
Sf1-Rspo3GOF
Control
Sf1-Rspo3GOF
C
Control
Sf1-Rspo3GOF
♀
♂
BrdU/DAPI
D
Control
Sf1-Rspo3GOF
♂
Rspo3

Fig. S6. Ectopic RSPO3 expression results in female-specific hyperplasia. A) Schematic representation of the genetic strategy to overexpress RSPO3 in adrenocortical cells using Sf1-Cre. B) Mean adrenal weight normalized to total body weight from 6 week and 3-month-old male andfemale control or *Sf1-Rspo3*^{GOF} adrenals. Error bars represent SEM. The numbers below the graph columns represent number of samples in each group (n). Statistical analysis for 6 weeks was conducted using Welch's one-way ANOVA followed by the Dunnett's T3 multiple comparisons test. Adjusted P-values: Control F vs *Sf1-Rspo3*^{GOF} F: P= 0.0008, Control F vs Control M: P=0.0001, *Sf1-Rspo3*^{GOF} F vs *Sf1-Rspo3*^{GOF} M: P=0.0043, Control M vs *Sf1-Rspo3*^{GOF} M: P=0.0130. Statistical analysis for 3 months was conducted with one-way standard ANOVA followed by Tukey's post-hoc test. Adjusted P-values: Control F vs *Sf1-Rspo3*^{GOF} F: P=0.0039, *Sf1-Rspo3*^{GOF} F vs *Sf1-Rspo3*^{GOF} M: P=0.0102, Control M vs *Sf1-Rspo3*^{GOF} M: P=0.9910. C) BrdU analysis to label proliferative cells in control and *Sf1-Rspo3*^{GOF} adrenal at 6 weeks of age. D) In situ hybridization for *Rspo3* using the RNA Scope method (*Rspo3* mRNA shown as red dots). Representative images represent control and *Sf1-Rspo3*^{GOF} adrenals from male 6-week-old animals. Scale bars: 50 μm.

Disease Models & Mechanisms • Supplementary information

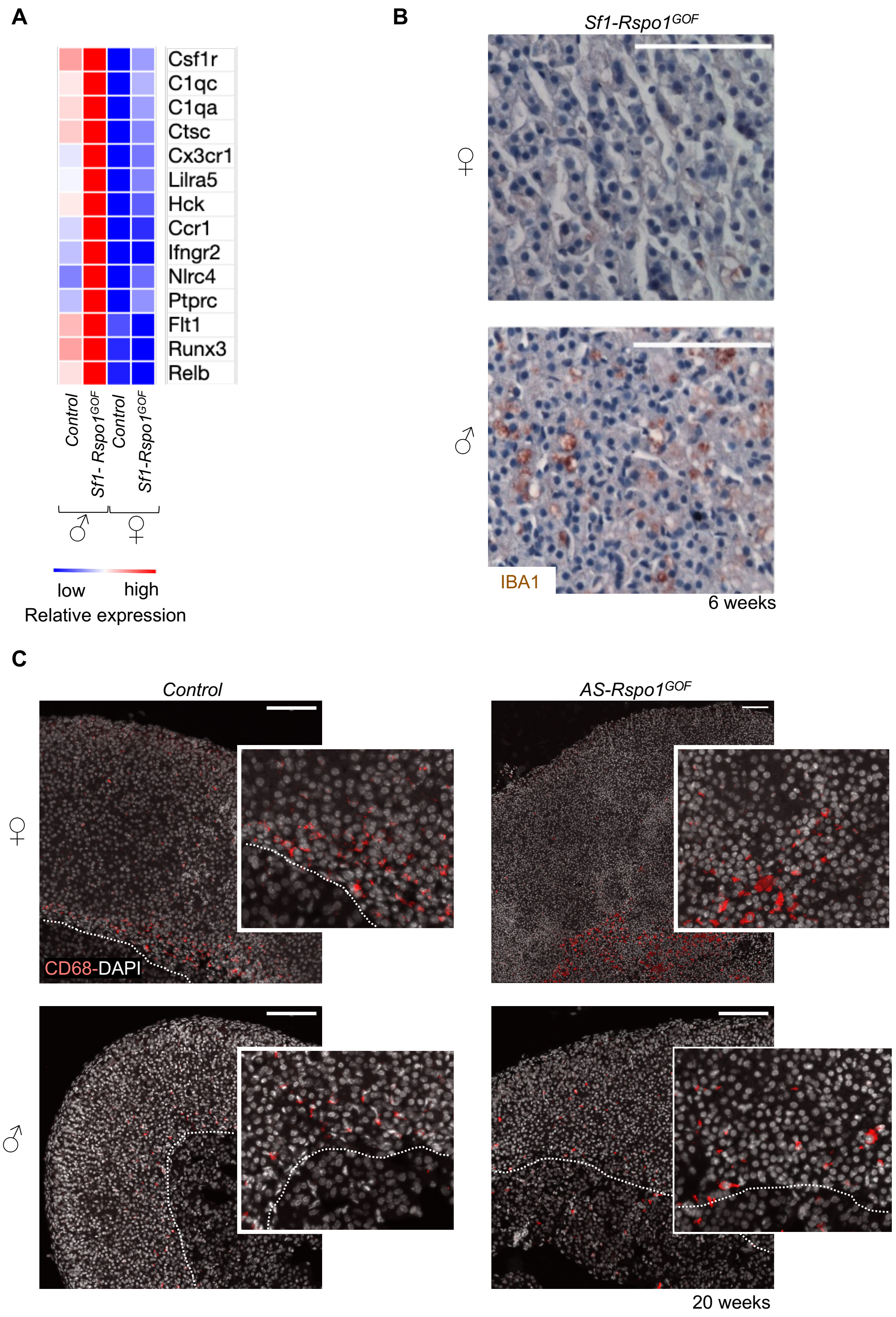

Disease Models & Mechanisms • Supplementary information

Fig. S7. Degenerative lesion formation by macrophages in the male *Sf1-Rspo1*^{GOF} adrenal cortex. A) Heatmap representation of relative expression differences (shown in color code) for genes expressed in macrophages/monocytes or pan-immune cells. B) Immunohistochemical staining for IBA1, a macrophage marker enriched in StAR deficient adrenals. Scale bar: 50 µm. C) Immunofluorescence staining for macrophage marker CD68 on 20-week-old adrenal cortex from control and *AS-Rspo1*^{GOF} animals. Scale bar: 100 µm.

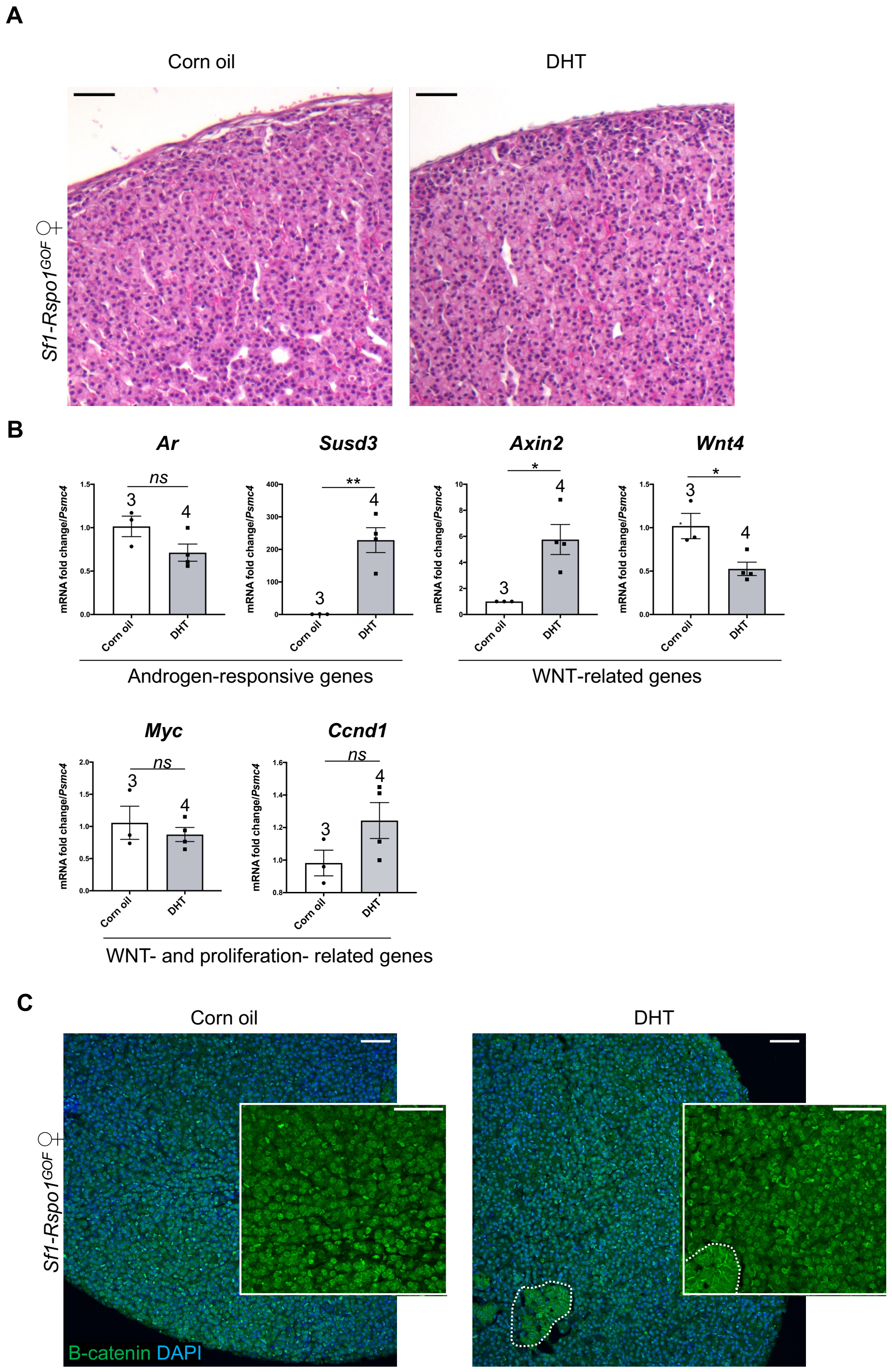

Disease Models & Mechanisms • Supplementary information

Fig. S8. Effect of DHT treatment on histology and expression in *Sf1-Rspo1*^{GOF} adrenals.

A) H&E staining on adrenal sections from 6-week-old female mice treated with corn oil or DHT during puberty. Scale bar: 50 µm. B) RT-qPCR analysis of gene expression for WNT signaling target genes (*Wnt4*, *Axin2*), known target genes of WNT signaling also related to proliferation (*Myc*, *Ccnd1*), and genes possibly related to androgen receptor signaling (*Ar*, *Susd3*). Graphs represent mean fold expression change comparing corn oil to DHT treated adrenals (normalized to *Psmc4* expression). Error bars represent SEM. The numbers above the graph columns represent number of samples in each group (n). Statistical analysis was conducted with unpaired t-test. P values: 0.1056 (*Ar*), 0.0173 (*Axin2*), 0.0228 (*Wnt4*), 0.0040 (*Susd3*), 0.5019 (*Myc*), 0.1347 (*Ccnd1*). DHT: dihydrotestosterone. C) Immunofluorescence staining for β-catenin on adrenal sections from 6-week-old female mice treated with corn oil or DHT during puberty. Inserts represent zoomed-in areas of the inner cortex. Scale bar: 100 µm

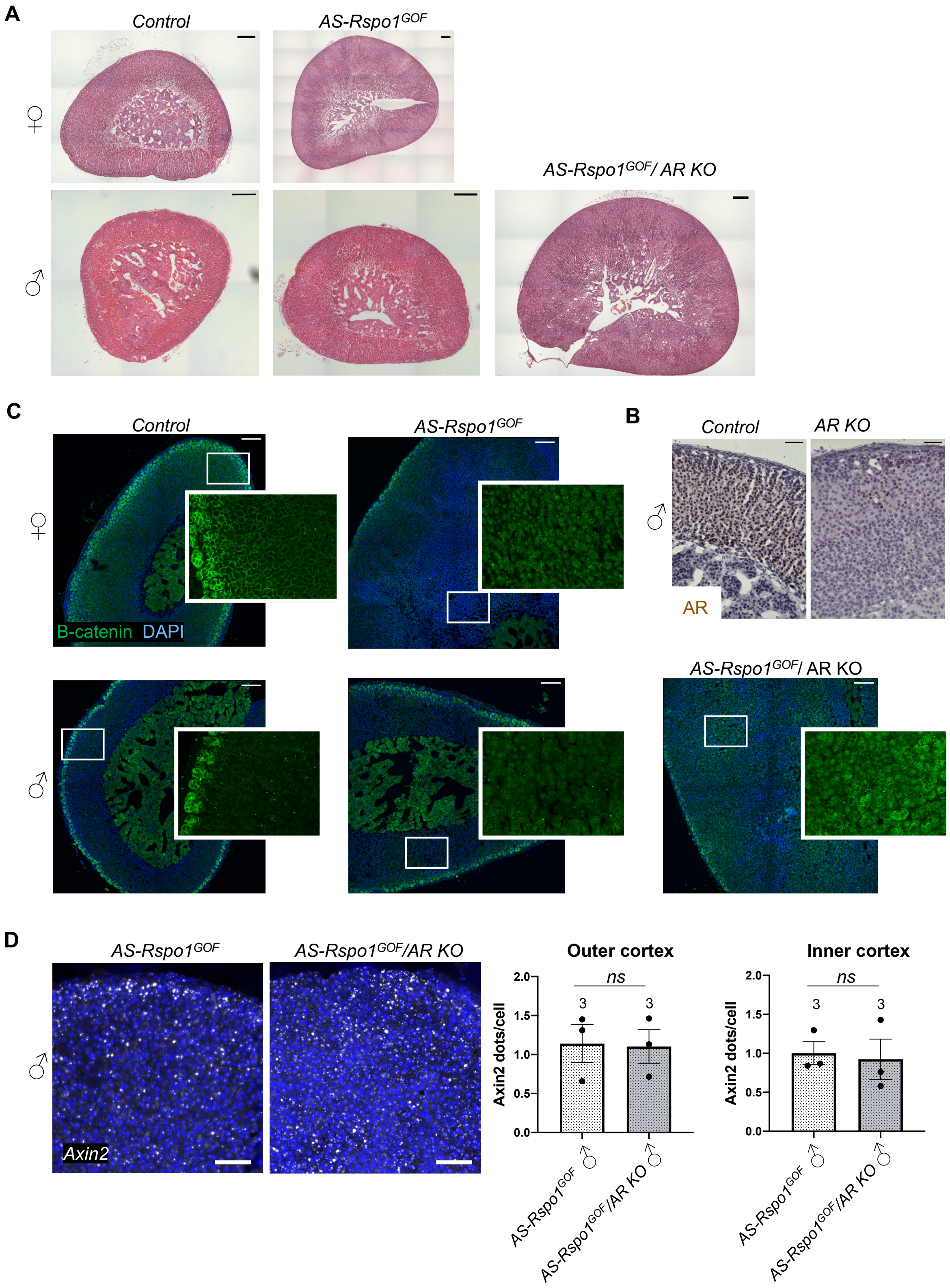

Disease Models & Mechanisms • Supplementary information

Fig. S9. Effect of *Ar* knock-out in adrenocortical histology, AR expression, and WNT signaling. A) Hematoxylin and eosin staining of adrenal sections at 20 weeks of age. Scale bar: 200 µm. B) Representative images of immunohistochemistry to detect AR in control and *Ar KO* male adrenals at 20 weeks of age. White arrows point to a few nuclei that display residual expression of AR in the *Ar KO* adrenals. Scale bar: 50 µm. C) Immunofluorescence staining for β-catenin on adrenal sections at 20 weeks of age. Inserts represent zoomed-in areas of the inner cortex. Scale bars: 100 µm. D) Quantification of *Axin2* expression on sections from *AS-Rspo1*^{GOF} and *AS-Rspo1*^{GOF}*/AR KO* male adrenals using RNA Scope single-molecule (ISH). The numbers above the graph columns represent number of samples in each group (n=3). Two-tailed unpaired t-test was used to determine statistical significance. Scale bars: 50 µm.

Disease Models & Mechanisms • Supplementary information

Table S1. List of primers used in this study.

Gene	Forward primer	Reverse primer 1	Reverse primer 2	Purpose
Psmc4	TTCTTGGAAGCTGTGG ATCA	TCAGGATGCGCACATAATA GTT		qPCR
Rspo1	CAGTGACTATGCGGCT TGG	AGAGCTCACAGCCCTTGG		qPCR
Susd3	GACTGTGCTCATATTCC ACTGC	TAAAGCCGAAGGTCTCAT GC		qPCR
Ar	GTGCCCACCCAAAGGA CT	CCTTTTCCCAATGCCTCAG		qPCR
Pcna	CTAGCCATGGGCGTGA AC	GAATACTAGTGCTAAGGT GTCTGCAT		qPCR
Pola1	TGCACTGTGGATAAAT TCTCAAG	CAGTGAGGTTTGACCCAT CC		qPCR
Cdc6	TCCGTGTGTGGACGTA AAAC	GGAGTGTTGCACAGGTTG TC		qPCR
E2f8	CCAGAAATCAGCCCAA ACA	GCAGACTGCTCAGCCTCT AAG		qPCR
Ccnd1	GCAGAAGGAGATTGTG CCATCC	AGGAAGCGGTCCAGGTAG TTCA		qPCR
Myc	CCTAGTGCTGCATGAG GA GA	TCCACAGACACCACATCA ATTT		qPCR
Rspo1 transgene	TGTTCATGTCGGGGTT GCGG	CGACCTGCAGCCCAAGCT AG		Genotyping
Rosa26	AGGGAGCTGCAGTGG AGTAG	AGCCTGCCCAGAAGACTC CC		Genotyping
Sf1-Cre	CCCACCGTCAGTACGT GAGATATC	CGCGGTCTGGCAGTAAAA ACTAT		Genotyping
Rspo3 transgene	CGCGATTAAATCGATCC CG	CCTATCTGCTTCATGCCAA TCC		Genotyping
Wt1-Sox9 transgene	CATCCGAGCCGCACCT CATG	GCTGGAGCCGTTGACGCG		Genotyping
Cyp11b2-Cre	GAGCTGGGGCCCATTT TCAGG	GCTCCAGGTGCATCCGAC GG	AACTTGCACCAT GCCGCCCA	Genotyping
Sry	TTGTCTAGAGAGCATG GAGGGCCATGTCAA	AACTTGCACCATGCCGCC CA		Genotyping
Ar flox	AGCCTGTATACTCAGTT GGGG	AATGCATCACATTAAGTTG ATACC		Genotyping

Disease Models & Mechanisms • Supplementary information

Table S2. List of antibodies used in this study.

**Primary antibodies**	**Source**	**Reference**	**Dilution**
Anti-Tyrosine hydroxylase (TH)	Sigma-Aldrich	AB152	1:200
Anti-CYP21A2	Sigma-Aldrich	HPA048979	1:200
Anti-AKR1B7 (M13)	Santa Cruz	sc-27763	1:200
Anti-3BHSD (P18)	Santa Cruz	sc-30820	1 :200
Anti-AR	Abcam	ab108341	1 :200
Anti-IBA1	Proteintech	10904-1-AP	1 :200
Anti-CD68	Proteintech	28058-1-AP	1:2000
Anti-DAB2 (E-11)	Santa Cruz	sc136964	1:50
Anti-BrdU (3D4)	BD Pharmingen	555627	1:100
**Secondary antibodies**	**Source**	**Reference**	**Dilution**
Donkey anti-rabbit Alexa Fluor 647	Invitrogen	A31573	1:400
Donkey anti-goat 555	Invitrogen	A21432	1:400
Donkey anti-mouse Alexa Fluor 555	Invitrogen	A31570	1:400
Biotinylated donkey anti-rabbit	Jackson Immunoresearch	711.065.152	1:400
Donkey anti-rabbit Alexa Fluor 555	Invitrogen	A31572	1:400
Donkey anti-mouse Alexa Fluor 647	Invitrogen	A31571	1:400

Disease Models & Mechanisms • Supplementary information
